# Intensifying Cyclopentanone Synthesis from Furfural Using Supported Copper Catalysts

**DOI:** 10.1002/cssc.202401484

**Published:** 2024-11-08

**Authors:** Adarsh Patil, Maurik Engelbert van Bevervoorde, Fernanda Neira d'Angelo

**Affiliations:** ^1^ Sustainable Process Engineering Group Eindhoven University of Technology P.O. Box 513 5600 MB Eindhoven The Netherlands

**Keywords:** Hydrogenation, Furfural, Cyclopentanone, Copper catalysis, Polymerization

## Abstract

This work addresses catalytic strategies to intensify the synthesis of cyclopentanone, a bio‐based platform chemical and a potential SAF precursor, via Cu‐catalyzed furfural hydrogenation in aqueous media. When performed in a single step, using either uniform or staged catalytic bed configuration, high temperature and hydrogen pressures (180 °C and 38 bar) are necessary for maximum CPO yields (37 and 49 %, respectively). Parallel furanic ring hydrogenation of furfural and polymerisation of intermediates, namely furfuryl alcohol (FFA), limit CPO yields. Employing a two step configuration with optimal catalyst bed can curb this limitation. First, the furanic ring hydrogenation can be suppressed by using milder conditions (i. e., 150 °C and 7 bar, and 14 seconds of residence time). Second, FFA hydrogenation using tandem catalysis, i. e., a mix of β‐zeolite and Cu/ZrO_2_, at 180 °C, 38 bar and 0.6, allows sufficient time for CPO formation and minimises polymerisation of FFA, thereby resulting in 60 % CPO yield. Therefore, this work recommends a split strategy to produce CPO from furfural. Such modularity may aid in addressing flexible market needs.

## Graphical abstract

The furfural to cyclopentanone reaction network exhibits varying reaction conditions for each step. This disparity makes it difficult to achieve selective conversion to cyclopentanone in a single step. Therefore, a two‐step strategy is proposed to optimize cyclopentanone yields.

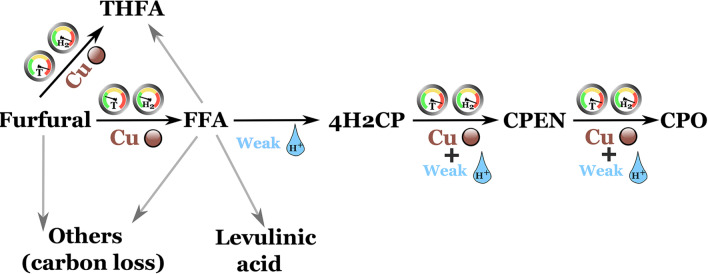

## Introduction

Replacing fossil fuels with renewable feedstocks such as biomass has attracted attention in recent years. Valorization of biomass can help reduce carbon footprint of raw materials‐to‐finished goods while meeting growing demands of chemicals and fuel. In 2004, the Department of Energy (DoE) included furfural as a potential chemical building block that can be obtained from the hemicellulose fraction of biomass.^[1]^ Synthesising furfural from hemicellulose and its derivative sugars (xylose, arabinose) has been done for more than a century, based on the process pioneered by the QUAKER OATS.^[2]^ Further, furfural can be converted into a diverse set of chemicals including furfuryl alcohol (FFA) for polymeric resins and foundry binders; tetrahydrofurfuryl alcohol (THFA), used for manufacturing dyes for leather, rubber and nylon; 1,5 pentanediol (1,5‐PeD) as plasticizer for adhesives; cyclopentanone (CPO) as flavor, fragrance and more recently as a jet fuel precursor.^[3]^

Conventional and recent alternatives for CPO production still use fossil‐based intermediates like adipic acid or dicyclopentadiene obtained from naphtha cracking.[^4^, ^5^, ^6^] Besides their fossil‐based origins, these processes suffer from high energy consumption and difficult separation and handling steps. Hence, a more sustainable bio‐based CPO synthesis method is highly desirable. In 2012, Hronec et al. reported the aqueous phase hydrogenation of furfural to CPO in presence of noble metal catalysts such as Pd, Pt and Ru supported on activated carbon.^[7]^ Since then, numerous works have reported CPO synthesis from furfural/FFA using (non) noble metals supported on different acidic/basic supports in a variety of solvent systems and a range of H_2_ pressures. Nevertheless, while it is evident that the hydrogenation of furfural yields a wide spectrum of products depending on the catalyst of choice, reaction conditions and solvents, controlling the selectivity towards targeted products remains a key challenge.

**Figure 1:**
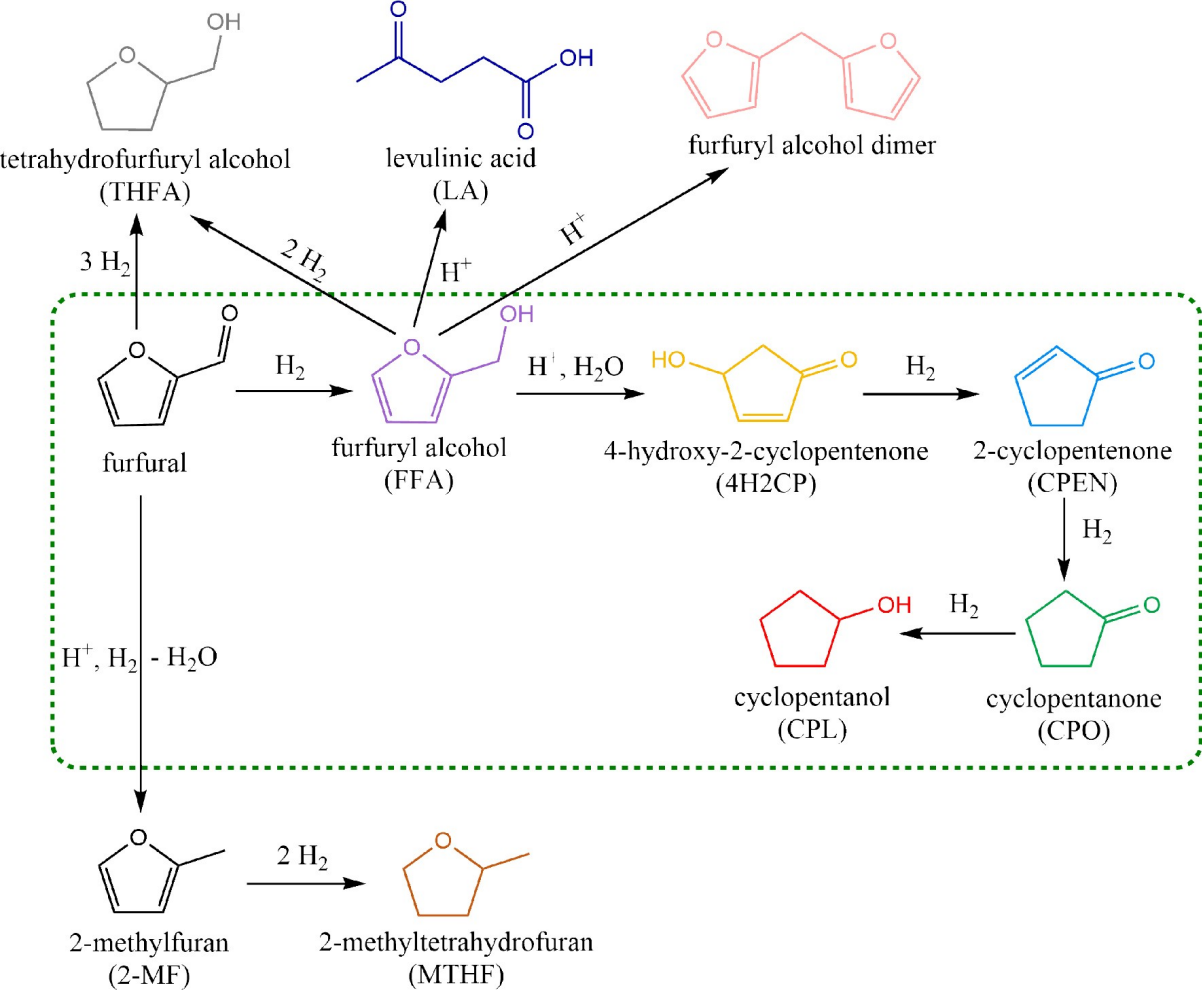
Reaction scheme for hydrogenation of furfural to CPO as collated from literature. The green box indicates the preferred reaction network of furfural hydrogenation to cyclopentanone.

Relatively high yields (~60–85 %) of CPO have been reported from furfural hydrogenation using both noble metals (e. g., Pt/C,^[7]^ Pd/f‐SiO_2_ ^[8]^ or even mixtures of Ru/C and ${\mathrm{Al}}_{11.6}{\mathrm{PO}}_{23.7}$ ^[9]^) and non‐noble metal catalysts (e. g., commercial nickel catalysts such as G‐134A and Ni‐SAT 320RS from Süd Chemie,^[10]^ Ni‐NiO/TiO_2_ ^[11]^) at temperatures around 140–190 °C and 15–80 bar H_2_ pressure. Although CPO can be synthesized from a diverse range of catalysts over a range of temperature and pressure, the choice of solvent primarily remains the same, i. e., water. Any deviation in this choice results in a decrease of selectivity of CPO from furfural hydrogenation.[^10^, ^12^, ^13^, ^14^, ^15^] For example, when using Ru/C and ${\mathrm{Al}}_{11.6}{\mathrm{PO}}_{23.7}$ in presence of tetrahydrofuran (THF) and methanol as solvent, the selectivity towards CPO dropped to zero, with THFA as the dominant product (yield >90 %). Alternatively, starting with a mixture of n‐butanol and water (1 : 1 vol.) rather than pure water switched the selectivity of products to 2‐methylfuran (2‐MF) as opposed to CPO.^[9]^ Based on the interest in controlling product selectivity during furfural hydrogenation, the study of the reaction mechanism remains a topic of great interest. The majority of the open literature concludes that the first hydrogenation step of furfural to FFA is the fastest step in the reaction network (Figure 1).[^16^, ^17^] Yet, several explanations have been proposed for the subsequent rearrangement and hydrogenation steps for the conversion of FFA to CPO. A significant body of literature suggests that Lewis acids favour the rearrangement of FFA to 4‐hydroxy‐2‐cyclopentenone (4H2CP).[^18^, ^19^, ^20^, ^21^, ^22^] On the other hand, Zhou et al.^[23]^ recently proposed a different route based on basic supports such as MgO/Al_2_O_3_, as they were deemed useful to prevent degradation of FFA via polymerization. Nevertheless, the role of supports in the transformation of furfural and/or its intermediate species towards CPO under aqueous phase conditions remains a subject of investigation. From a process implementation perspective, there is great interest in developing continuous and stable catalytic processes that present well‐known techno‐economic advantages over the batch‐counterparts (e. g., limited downtime, better integration of process streams and heat flows.) With this respect, resolving stability issues during continuous catalytic biomass conversion remains a key challenge.

Thus, this work delves into the to‐date unresolved effects of the catalyst support on the various reaction steps in the conversion from furfural to CPO, namely the hydrogenation of furfural to FFA, the rearrangement of FFA and the subsequent hydrogenation steps to CPO (Figure 1). The acidic properties of supported Cu catalysts with varying acidity (i. e., ZrO_2_, ZnO‐Al_2_O_3_ and MgO‐Al_2_O_3_), as well as physical mixtures of selected Cu‐catalysts with β‐zeolite are investigated. To that end, the work employs a continuous flow reactor to perform parametric studies such as variations of catalysts type and operating conditions (i. e., feed flow rates, temperature and pressure). Based on this understanding, we further rationalize and optimize the rate‐limiting steps in the synthesis of CPO. Importantly, we show that the single‐step synthesis of CPO from furfural is not optimal. For the first time, the work proposes a two‐step catalytic strategy as an attractive method for the conversion of furfural to CPO. Leveraging the benefits of flow‐through reactor systems, this study sheds light into long‐term stability of this non‐noble metal catalyst. To the best of our knowledge, these insights have not been reported in the literature yet.

## Results and Discussion

### Catalyst Characterisation

Table 1 summarizes the most important properties of the catalyst samples used in this work. The surface area of all three supported Cu catalysts are comparable with each other. Additionally, the Cu/ZrO_2_ surface area is similar to that observed by Zhang et al.^[24]^ All of the catalysts exhibit IUPAC Type IVa adsorption isotherms accompanied by hysteresis, which is typical of mesoporous materials (see Figure S5 in SI). The hysteresis loops on all three catalysts resemble a bean‐pod shape with the loop closure at p/p_0_ ~0.5. Therefore, the pores can be a mix of well‐ordered mesopores of tubular shape and macropores that are not completely filled by N_2_ during the measurement.[^25^, ^26^] Among the three catalyst samples, Cu/MgO‐Al_2_O_3_ has the highest fraction of mesopores, as seen by a steep decrease in the desoprtion branch at p/p_0_ ~0.7, and confirmed by a well‐defined peak at ~8 nm in the pore size distribution curve (see Figure S6 in SI). Both Cu/ZnO‐Al_2_O_3_ and Cu/ZrO_2_ follow a more traditional H3 hysteresis loop. β‐zeolite (Si : Al ‐ 25) used in this work exhibits a typical Type I isotherm indicative of microporous structure of the catalyst. Besides these physical properties, the composition of the three supported‐Cu catalysts is depicted in Table S1. The Cu loading (measured using SEM‐EDX) for the synthesized Cu/ZrO_2_ and Cu/MgO‐Al_2_O_3_ catalysts are approximately, 29.4 and 24 wt.% respectively. Although the Cu loading for the hydrotalcite support is lower than the nominal loading (i. e., 30 wt.%), no blue hue was noted in the centrifuged liquid after catalyst precipitation. The atomic ratio of Mg : Al was found to be 3 and agreeable to the nominal atomic ratio. Cu/ZnO‐Al_2_O_3_ exhibits the finest Cu^0^ (111) crystallite size among the three catalysts, followed by Cu/MgO‐Al_2_O_3_ and Cu/ZrO_2_. The exposed Cu surface area obtained by 2‐mercaptobenzimidiazole (MBI) titration^[27]^ showed no significant difference. XRD analysis of the reduced and passivated catalyst samples (Figure S1 in SI) shows a distinct peak for Cu(111) cystallographic plane with other planes such as Cu(200) and Cu(220), vide infra JCPDS card number 04‐836. Additionally, the appearance of Cu_2_O (111) reflection for the reduced catalysts is an indication of the final passivation step on the catalyst surface while preserving metallic Cu in the bulk of catalyst in its reduced form. These results demonstrate the effectiveness of catalyst preparation protocol, including reduction and passivation ex‐situ. It should be noted that the XRD analyses were done approximately five to six hours after the final passivation step with continuous exposure to ambient air. The size of Cu^0^(111) crystallite is higher for both the ZrO_2_ and MgO‐Al_2_O_3_ than the commercial Cu catalyst as depicted in Table 1. This is in contrast with loading (30 wt.% nominal) of Cu in the synthesized catalysts as compared to 55 wt.% for the commercial catalyst. Traditionally, the commercial Cu/ZnO‐Al_2_O_3_ catalyst is synthesized via co‐precipitation method and the catalysts synthesized in this work use NaBH_4_ as the reducing agent in solvent conditions. NaBH_4_ being a strong reducing agent might be the cause for a larger crystallite size for Cu when compared with that for commercial Cu catalyst. TPR analysis of the Cu catalysts (Figure S8 in SI) reveals that all samples are reduced below 300 °C, which is the temperature chosen for the reduction protocol. Commercial Cu/ZnO‐Al_2_O_3_ and synthesized Cu/MgO‐Al_2_O_3_ exhibit complete reduction up to temperatures ~200 °C. For the former case, Bokhoven et al., demonstrated that Cu present in the commercial catalyst displays different reduction behaviour at different pressures (ranging from 1 mbar to 10 bar) with full Cu reduction from +2 to metallic (0) state before 200 °C at 1 bar.^[28]^ A similar TPR curve is observed with the hydrotalcite (MgO‐Al_2_O_3_) support accompanied by a minor shoulder between 200 and 220 °C. The absence of peaks at T>300 °C indicates the absence of bulk Cu species such as CuAl_2_O_4_.[^29^, ^30^] Absence of crystalline peaks for the spinel structure in the XRD pattern of Cu/MgO‐Al_2_O_3_ (see Figure S1 in SI) supports this finding. The zirconia‐supported Cu displays a broader reduction profile. This can be attributed to the formation of Cu‐O−Zr like structure as observed by Zhang et al.^[24]^

**Table 1:** Compositional and structural properties of the catalysts.

Catalyst	S_BET_ 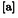	Av. pore size	Cu loading	Cu^0^ surface	Crystallite size	Acidity
	m^2^ g^−1^	nm	wt.%	m^2^ g^−1^	nm^[b]^	mmol H^+^ g^−1^ 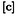
Cu/ZrO_2_	94.05	6.18	29.36	14.54	33.0	0.35 (0.24)
Cu/ZnO‐Al_2_O_3_	99.8	8.57	54.08	18.10	12.5	1.2 (0.12)
Cu/MgO‐Al_2_O_3_	142.7	8.1	24.00	17.36	21.8	1.2 (0.09)
β‐zeolite	441.14	2.41	N.A.	N.A.	N.A.	1.1 (0.21)

[a] Surface area by BET; [b] Cu^0^ (111) crystallographic plane; [c] In parenthesis, density of weak acid sites measured by NH_3_‐TPD.

**Figure 2:**
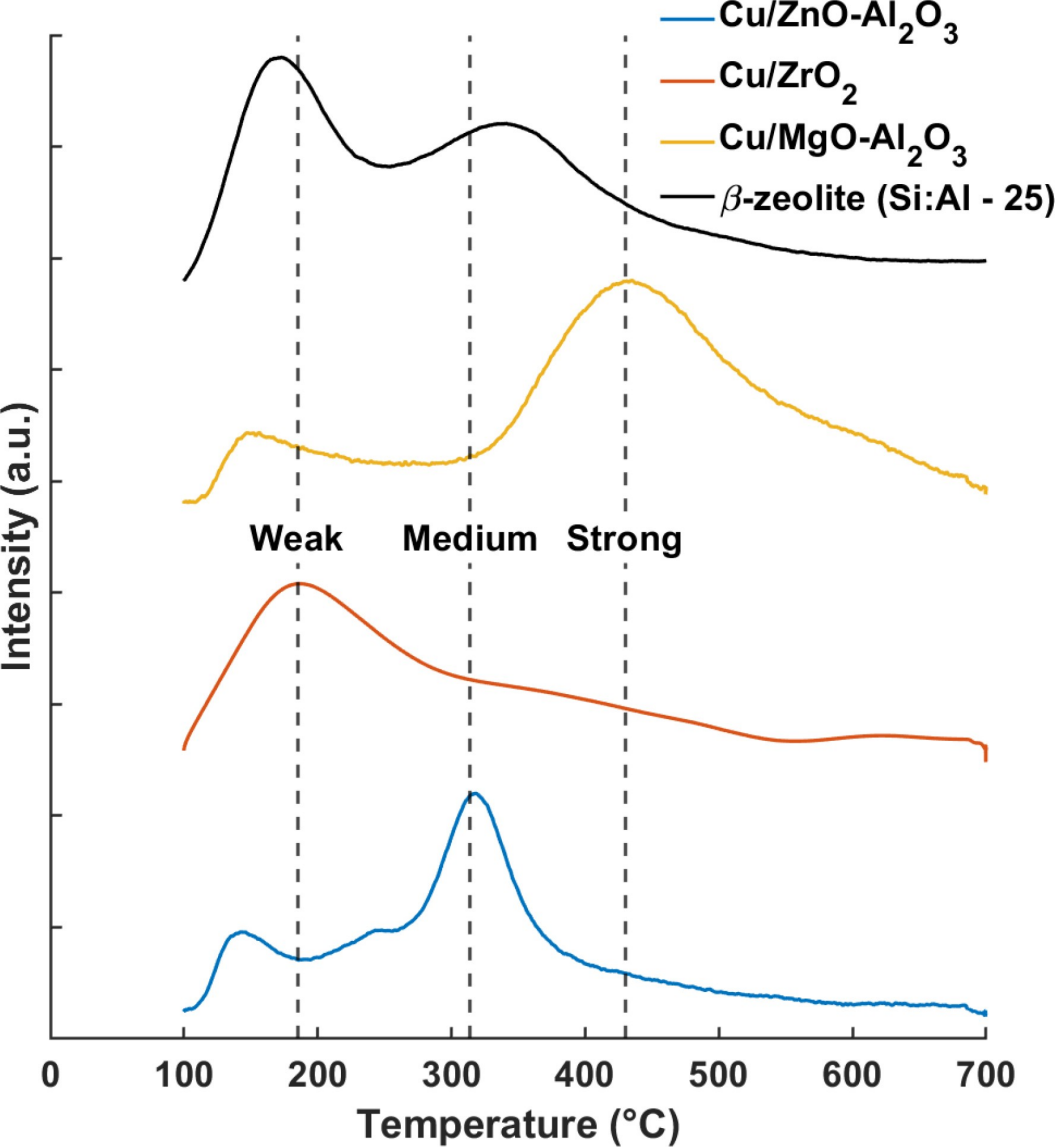
NH_3_‐TPD of the different supported‐Cu catalysts.

Investigation into the acidity of the catalyst was done using NH_3_ TPD (Figure 2). Cu/ZrO_2_ presents the highest fraction of weaker (in this case, Lewis) acid sites as compared to the other two Cu catalysts. Similar findings were reported by Zhang et al. using identical catalyst synthesis procedure. The presence of Cu^+^−O−Zr structure created the Lewis acidity, confirmed with pyridine FT‐IR analysis.^[24]^ The catalysts containing alumina possess greater proportion of either medium and high strength acid sites, lacking in low strength acidity. Surprisingly, the Cu/MgO‐Al_2_O_3_ catalyst, which was supposed to posses basic sites, instead exhibits acidic character, with high strength acid sites (desorption temperature for NH_3_ >420 °C). β‐zeolite exhibits a mix of weak and medium strength acid sites with the former ascribed mostly to Lewis sites and weak Brønsted sites.[^31^, ^32^]

### Furfural Hydrogenation Over Supported‐Cu Catalysts

Commercial Cu/ZnO‐Al_2_O_3_ was chosen as the benchmark catalyst to explore furfural hydrogenation with different feed contacting (i. e., residence times) at 150 °C as depicted in Table 2. The results summarized in Table 2 show full furfural conversion under all WHSV conditions explored, while there is an evident variation in FFA selectivity and carbon balance upon changing WHSV and liquid residence times. Entry 1 in Table 2, bearing the highest contacting between the catalyst and furfural feed (i. e., lowest WHSV), also exhibits the greatest carbon loss. Decreasing the contacting times ten‐fold (i. e., increasing WHSV to $0.6\;g_{\mathrm{furfural}}\;g_{\mathrm{cat}}^{-1}\;{\mathrm{hr}}^{-1}$ , shown in Entry 4) effectively renders selective FFA production from furfural with minimal carbon loss. This confirms that furfural degradation can be disregarded under these conditions (i. e., 150 °C, 12 bar, Cu/ZnO‐Al_2_O_3_) and that carbon unbalance encountered under extended residence times is due to undesired reactions from FFA or its derivatives. Entry 2 shows an intermediate scenario where the contacting time is sufficiently large to selectively produce and further convert FFA into potentially attractive products (e. g., CPO or CPO precursors), but not as long as to cause severe degradation. Such conditions render small amounts of hydrogenation and rearrangement products, albeit at the expense of increasing carbon losses. For example, using a WHSV of 0.24 g_furfural_ g_cat_^‐1^ hr^−1^ results in a drop of FFA selectivity to 94.7 % (ca. 5 % FFA is converted away), only 2 % of valuable products, and a 3 % of carbon unbalance. Alternatively, extending the liquid residence time while preserving the WHSV (entry 3) results in further drop in FFA selectivity and major drops in carbon balance.

**Table 2:** Effect of catalyst contacting on furfural conversion to FFA using 1 wt.% furfural in water. Conditions: T=150 °C; P_total_=12 bar; Catalyst=Commercial CZA; W_cat_=1 gram; W_SiC_=3 grams.

WHSV	*τ*	X_furfural_	$S_{\mathrm{FFA}}$	$S_{\mathrm{FFA}\;\mathrm{derivatives}}$ 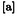	Carbon balance
$g_{\mathrm{furfural}}\;g_{\mathrm{cat}}^{-1}\;{\mathrm{hr}}^{-1}$	seconds	%	%	%	%
0.06	138	100	46.7	24.2	71.9
0.24	35	100	94.7	3.2	97.8
0.24^[b]^	69	100	54.7	21.6	76.1
0.6	14	100	99.5	0.5	100

[a] Sum of FFA, 4H2CP, CPEN and CPO yields. [b] Achieved by halving catalysts load and flow rates, thus doubling liquid residence time.

Since furfural hydrogenation is a rather fast reaction, the likelihood of intra‐particle diffusion effects on the observed furfural hydrogenation rates was investigated via Weisz‐Prater (WP) criterion. The WP number was evaluated for 53–80 μm catalyst particle size based on the maximum feed flow rate and conversion, as it represents the highest reaction rate. Under these conditions, a WP number of 0.035 (less than 0.4) confirms the absence of internal mass transfer limitations within the catalyst particles used in this work. It is therefore possible to selectively and effectively produce FFA from furfural at 150 °C and 12 bar of total pressure with relatively short contact times (WHSV of $0.6\;g_{\mathrm{furfural}}\;g_{\mathrm{cat}}^{-1}\;{\mathrm{hr}}^{-1}$ , entry 4) under kinetic regime.

**Figure 3:**
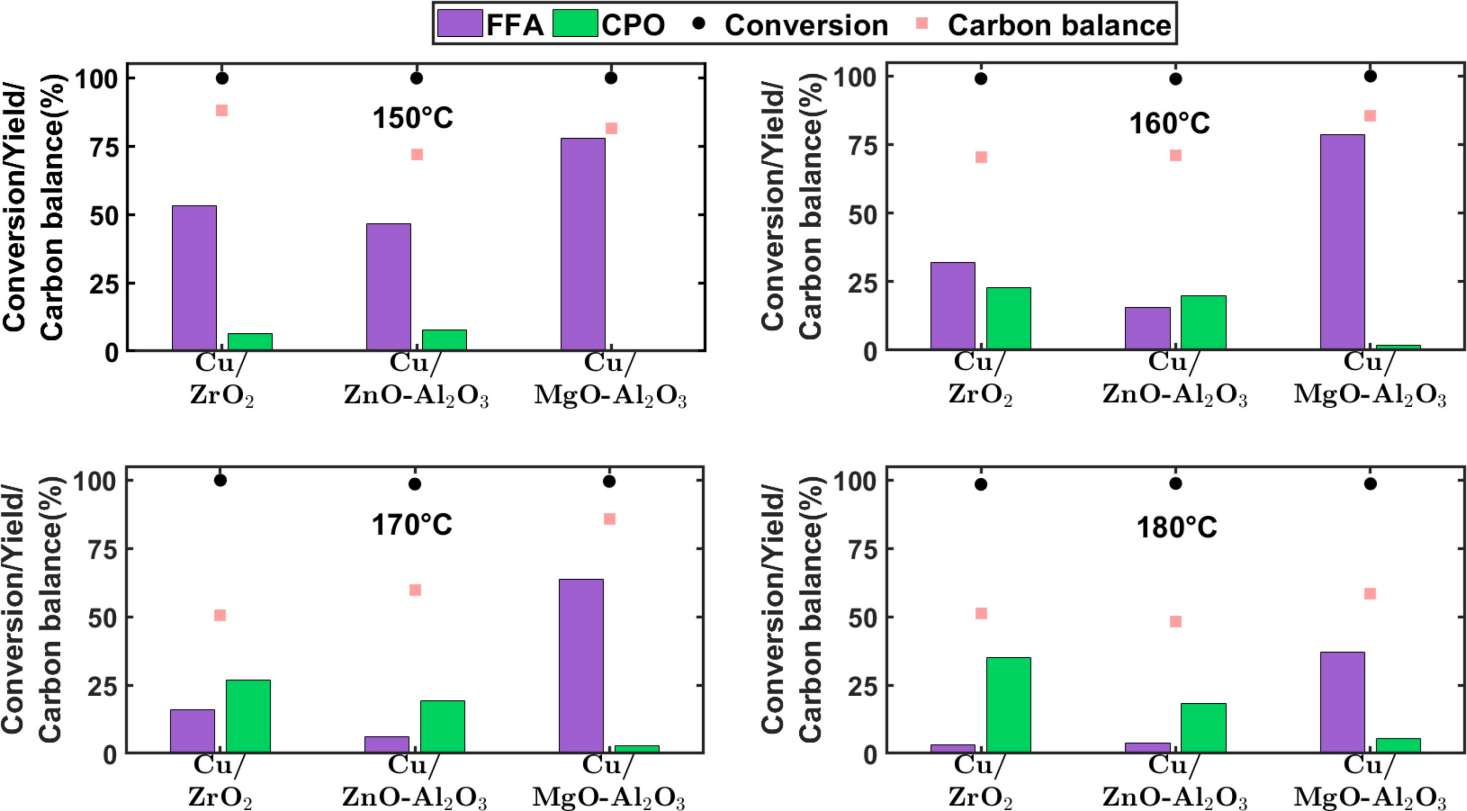
Comparison of different supports on furfural hydrogenation to cyclopentanone using 1 wt.% furfural in water. Conditions: T=150–180 °C; P_total_=12 bar; W_cat_=1 gram; W_SiC_=3 gram; Q_feed_=0.1 mL min^−1^, Q_H2_=5 NmL min^−1^; and WHSV=$0.06\;g_{\mathrm{furfural}}\;g_{\mathrm{cat}}^{-1}\;{\mathrm{hr}}^{-1}$ .

In view of conflicting hypotheses underlying the role of supports for transforming FFA to the desired rearrangement pathway, different acidic supports were used to enable furfural transformation to CPO. Figure 3 shows the performance of different Cu‐catalysts with increasing acid strengths (left to right on the X axis) during furfural hydrogenation in the temperature range 150–180 °C (top‐left and bottom‐right subplots, respectively). The figure displays the conversion of furfural and yield to FFA and CPO as main products of interest (rest of the product/intermediates distribution shown in Figure S10 in SI), as well as the carbon balance. Furfural undergoes complete conversion with all the catalysts and temperatures investigated, irrespective of the nature of the supports. As seen in numerous works, regardless the active metals, furfural hydrogenation to FFA is the first and quickest step in the reaction network (Figure 1).[^16^, ^17^, ^33^] This is in line with the relatively high yields of FFA obtained for all catalyst tested in the lowest temperature range (150 and 160 °C). Parallel degradation of furfural under such conditions was proved to be minor in supporting experiments on ZrO_2_ (i. e., the catalyst support leading to the highest carbon losses), as reported in Table S2 in SI. Thus, incomplete carbon balance is mostly attributed to subsequent degradation of FFA, at least up to 160 °C.

Notwithstanding, the distribution of intermediates from FFA rearrangement, hydrogenolysis and hydrogenation steps is different for each catalysts. The hydrotalcite support (i. e., MgO‐Al_2_O_3_) exhibits highest acid strength among the three chosen supports. Despite this characteristic, relatively high proportion of FFA remains unconverted (78 and 37 % yield at 150 and 180 °C, respectively) across the entire temperature range. Products of FFA rearrangement and hydrogenation were detected in small amounts (see Figure S10), while CPO yields remain limited as well (i. e., 0.5 and 5 % at 160 and 180 °C, respectively). Other works have hypothesized the presence of basicity on MgO‐Al_2_O_3_ to be important in reducing the formation of polymeric species during furfural hydrogenation at 190 °C.^[23]^ The results in this work at 180 °C suggest otherwise. It is evident that FFA remains unconverted while operating at temperatures below 180 °C using MgO‐Al_2_O_3_ as the support. This is in line with Baldenhofer et al., who have demonstrated that neither FFA rearrangement to 4H2CP nor FFA self‐condensations to oligomers can be catalyzed merely by a homogeneous base in the form of NaOH (pH=10.5) at 160 °C.^[34]^ Therefore, catalyst basicity appears to be irrelevant for this reaction under the explored temperature range in aqueous conditions.

Decreasing the acid strength in the form of Cu/ZnO‐Al_2_O_3_ as the catalyst, results in a more favourable yields of FFA rearrangement products (including CPO) as compared to MgO‐Al_2_O_3_. The FFA yield drops to 16, 6 and 4 % at 160, 170 and 180 °C, respectively. The intermediates obtained upon FFA rearrangement (i. e., 4H2CP and CPEN) are observed in significant quantities (24 and 7, 20 and 4 %) while increasing temperature to 170 and 180 °C, respectively, albeit at the expense of higher carbon losses.

Further, employing ZrO_2_, i. e., a material possessing weak acidity, does selectively enable the rearrangement of FFA to 4H2CP, thereby rendering higher yield of CPO as compared to ZnO‐Al_2_O_3_ and MgO‐Al_2_O_3_ (i. e., catalyst with stronger acid sites) at all operating temperatures. This can be attributed to the ${\mathrm{Cu}}^{+}$ species exhibiting Lewis acidic character instead of the traditional Brønsted acidity arising from hydroxyl species on the catalyst surface.^[24]^ Other works using a Cu^[24]^ and a bimetallic NiFe catalyst^[13]^ report similar dependency of weak acid sites content on CPO yields. Additionally, minimal amounts of 4H2CP (Figure S10 in SI) is observed with this catalyst at T >150 °C, in contrast to the other materials tested. This suggests that the strength of acid sites plays a crucial role in hydrogenation of the intermediates to obtain CPO efficiently. Such observations are further supported by decreasing CPO yield with increasing acid strength for all temperatures used. Thus, Cu loading (30‐50 wt.% used in this work) and/or dispersion are not the sole factors that determine the hydrogenation activity of these catalysts with respect to intermediates (namely, 4H2CP, CPEN). Metal‐support interactions, as evidenced by a broadened peak of H_2_‐TPR profile up to 280 °C for Cu/ZrO_2_, may be of greater importance in enabling hydrogenation of intermediates.

Irrespective of the support used, carbon loss is the limiting factor to maximize CPO yields at higher temperatures. To further elucidate on the likelihood of furfural degradation as a source of carbon losses, additional experiments were performed at longest contact times (i. e., lowest WHSV using nitrogen in gas phase) using commercial Cu/ZnO‐Al_2_O_3_ at 170 and 180 °C (see Table S2 in SI); since Cu/ZnO‐Al_2_O_3_ exhibits ~40 and 50 % carbon loss at 170 and 180 °C. Considering the carbon loss resulting from furfural degradation and hydrogenation, it becomes clearer that significant losses originate from FFA rather than furfural. Recently, similar water soluble and insoluble oligomeric products with molecular weight >200 and >1000 g mol^−1^, respectively, were observed from FFA polymerization.^[34]^ It is evident that producing and further converting FFA by operating with extended contact times (i. e., lowering WHSV) under these conditions is challenged by severe FFA degradation, thus limiting the overall atomic efficiency of the process. In other words, a one‐pot conversion does not seem plausible without accepting major carbon losses. It is therefore recommended that the entire conversion from furfural to CPO be addressed in separate sequential catalytic steps with individually optimized conditions as discussed in the next subsections.

### Furfuryl Alcohol Rearrangement Over Acidic Catalysts

Regardless of the nature of support, the rearrangement of FFA to 4H2CP appears to the rate‐limiting step at 150 °C (top‐left subplot in Figure 3), as FFA remains the primary product. On increasing the temperature to 160 °C, this limitation is overcome for both the commercial Cu/ZnO‐Al_2_O_3_ and Cu/ZrO_2_ catalysts, as seen by the decrease of FFA yield from ca. 50 % to approximately 15 and 30 %, respectively. The presence of weak and moderate strength acid sites favour FFA conversion pathway towards CPO formation. However, the increase in temperature is accompanied with an increase in loss of total carbon yield in the products. Further increase in temperature to 180 °C exacerbates this problem. These findings suggest that FFA rearrangement is accompanied by a parallel degradation pathway leading to carbon loss, supported by the pool of products detected using GC‐MS (see Figure S14), and visual depiction of a sample obtained at 180 °C (in SI). Such products are predominantly in the range of 200–250 g mol^−1^ and they are possibly water soluble, as shown by Baldenhofer et al.^[34]^ Therefore, visual checks of the product sample do not necessarily reveal carbon deposits. Regardless, similar trends for carbon loss from molecules analogous to FFA 1‐(furan‐2‐yl)but‐2‐en‐1‐ol, (1‐(furan‐2‐yl)ethan‐1‐ol) have been reported by Ulbrich et al. using high temperature water and acetic acid as a catalyst^[35]^ with toluene as a co‐solvent. The authors demonstrate that low concentration of FFA in the feed is beneficial to obtain high 4H2CP yields. This is supported by an additional set of batch experiments conducted in this study (Figure S12 in SI). Moreover, similar findings were reported by Kumaraguru et al. by using NMP as a co‐solvent to suppress the formation of polymeric species, at the expense of lower catalytic activity.^[36]^ Hence, using relatively low operating temperature (ca. 150 °C) and supports exhibiting weak acidity enable selective FFA rearrangement towards 4H2CP and further hydrogenation to CPO, while higher temperatures leads to more carbon loss.

Given its relevance in the overall reaction scheme, we further investigated the rearrangement of FFA to 4H2CP. Figure S11 (in SI) shows the results of screening several acidic catalysts in a batch reactor at 150 °C for eight minutes of reaction time. Table 3 summarizes the FFA conversions and 4H2CP yields obtained upon exposure of aqueous solutions of FFA to the best performing catalyst (i. e., β‐zeolite and ZrO_2_) tested in flow configuration.

**Table 3:** FFA conversion to 4H2CP using 0.2 grams of catalyst with 1 wt.% FFA in water solution at 150 °C.

Catalyst	WHSV	*τ*	X_FFA_	$S_{4H2\mathrm{CP}}$
	$g_{\mathrm{FFA}}\;g_{\mathrm{cat}}^{-1}\;{\mathrm{hr}}^{-1}$	s	%	%
Blank 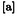	–	448	94.5	57.2
	0.6	45.2	99	70
	1.2	22.6	63.6	37.5
	0.6	14.2 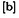	70	55
	1.2	7.1 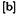	44.3	33.3
ZrO_2_ 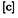	0.6	45.2	77.7	56.1

[a] No catalyst, only inert (4 grams SiC) [b] Under hydrogen flow [c] Commercial ZrO_2_

As depicted in Table 3, FFA rearrangement to 4H2CP takes place in absence of a catalyst, using merely inert SiC as the packing, at 150 °C. It takes about eight minutes of reaction time to undergo complete conversion. Further, packing the reactor with bare ZrO_2_ reduces 10‐fold the reaction time, indicating a catalytic effect. Using β‐zeolite, which possesses a higher density of weak acid sites as compared to bare ZrO_2_ (0.210 and 0.04 mmol H^+^g^−1^, respectively), further promotes the reaction rate (i. e., greater conversion at comparable reaction time). It should be noted that the selectivity towards 4H2CP increases with conversion for β‐zeolite, indicating that the rearrangement step must involve a sequential reaction scheme. Several works have hypothesized the formation of a carbocation on the aliphatic carbon of the furanic ring from the attack of water as a solvent.^[37]^ Equimolar addition of hydroxyl protecting species ((3‐Aminopropyl) trimethoxysilane), not only inhibits polymerization reactions, but also prevents FFA from undergoing any reaction (entry 6 in Table S3 in SI). This supports the aforementioned hypothesis. The formation of carbocation is then followed by either a rearrangement and ring opening of FFA to form 4H2CP, or a the formation of dimeric species resulting in further chain growth. Furthermore, different organic species in the form of solvents (Entries 1–4 in Table S3 in SI) were used to study their effects in increasing 4H2CP selectivity. Irrespective of the organic solvents used, no improvement in 4H2CP selectivity was observed. Thus, water is the optimal choice. With respect to catalysts, our results show that the selectivity towards 4H2CP is the highest with β‐zeolite. Increasing the Si : Al ratio of β‐zeolite from 25 to 360 reduced 4H2CP yield due to lower acid sites content. Hence, we hypothesize that a catalyst possessing weak acid sites promotes the formation of carbocation from FFA in presence of water, as well as 4H2CP selectivity, as opposed to catalysts with fewer weak acid sites such as ZrO_2_. A similar trend is followed in the case of H‐ZSM5 and Ga‐ZSM5 catalysts, with the latter exhibiting higher weak acid sites content (0.12 vs. 0.27 mmol H^+^g^−1^, respectively, see screening results Figure S9 in SI). Additionally, no significant effects were observed upon addition of hydrogen, besides the expected decrease in residence time and thus conversion. No hydrogenated products such as THFA or CPEN were observed in the GC analysis. Since formation of CPEN from 4H2CP is a hydrogenolysis (dehydration and hydrogenation) process, no intermediates such as 1,3 cyclopentadienone or 4‐hydroxy‐cyclopentanone were detected in either GC or GC‐MS.

### Furfuryl Alcohol Hydrogenation Over Cu/ZrO_2_ and β‐Zeolite Mixtures

Our findings so far point at the rearrangement of FFA to 4H2CP as one of the limiting steps in the entire conversion pathway from furfural to CPO at temperatures ca. 150 °C. Operating at greater temperatures may overcome such limitation but also results in important carbon losses. Among the different materials tested, β‐zeolite is the most efficient catalyst to ease the conversion of FFA to 4H2CP, while a hydrogenation catalyst is still required for the subsequent hydrogenation steps to CPO. Since the direct immobilization of Cu on β‐zeolite would render relatively low Cu^0^ loading and dispersion, possibly due to Cu incorporation in the zeolite framework as ${\mathrm{Cu}}^{2+}$ or ${\mathrm{Cu}}^{1+}$ ,[^38^, ^39^] thereby altering its original catalytic activity, this work uses a combination of β‐zeolite and Cu/ZrO_2_ to catalyze the conversion of FFA to CPO.

Upon exposure to hydrogenation and acidic conditions, FFA as substrate may undergo four possible parallel pathways (Figure 1). First, the desired pathway is the acid‐catalyzed rearrangement of FFA to form 4H2CP, which can be subsequently hydrogenated to CPO, and even further to cyclopentenone (CPEN). Secondly, in line with previous discussion, FFA may undergo polymerisation, likely catalyzed by acid sites, to form oligomeric species (di‐ or tri‐meric species detected in GC‐MS). The third possible pathway is the deep hydrogenation of the furanic ring to yield tetrahydrofurfuryl alcohol (THFA). Literature indicates the preferred orientation of FFA on the active Cu metal is through the furanic ring carbons, supported by theoretical calculations by Yao et al.,^[40]^ leaving the ‐OH group prone to attack from adjacent water molecules for rearrangement. Finally, FFA has been reported to suffer hydrolysis and ring opening to form levulinic acid in presence of H‐ZSM5 (i. e., an acid catalyst) at 90 °C.^[41]^ Thus, tuning acid strength and hydrogenation activity is important to control product selectivity.

**Figure 4:**
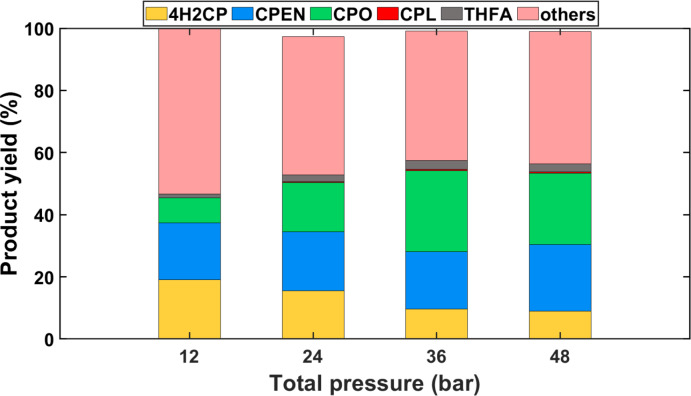
Effect of pressure on FFA hydrogenation to CPO using 1 wt.% FFA in water. Conditions: T=150 °C; W_cat_=0.2 grams of Cu/ZrO_2_ and β‐zeolite each; W_SiC_=3.6 grams; Q_feed_=0.2 mL min^−1^; $Q_{H_{2}}$ =4 NmL min^−1^, WHSV=0.6 $g_{\mathrm{FFA}}\;g_{\mathrm{cat}}^{-1}\;h^{-1}$ .

Figure 4 shows the product distribution obtained after FFA hydrogenation over a Cu/ZrO_2_ and β‐zeolite bed at 150 °C and various hydrogen pressures. It is evident that under these conditions, 4H2CP, CPO and CPEN (i. e., yellow, green and blue areas, respectively) are the primary products, along with other species (i. e., the pink areas) where the polymeric products are accounted for. Levulinic acid is not detected in the product mixture and THFA (denoted by the gray area in Figure 4) is produced in minor amounts. Thus, the first two reaction pathways (i. e., FFA rearrangement followed by hydrogenation, and polymerization) are likely the most significant in these conditions (using water as solvent). It should be noted that the reactions in Figure 4 were carried out under an order of magnitude shorter contact times than those in Figure 3 (i. e., WHSV 0.6 vs 0.06, respectively), where the hydrogenation of furfural rendered much lower CPO yields. This suggests that β‐zeolite is effectively catalyzing the rate limiting step, i. e. the rearrangement of FFA. Further, as shown in Figure 4, the product distribution shifts towards CPO and CPEN (i. e., hydrogenation products) when the hydrogen pressure increases, along with a decrease in yield to non‐quantified products. While greater hydrogen pressures favor the formation of THFA, it is evident that deep hydrogenation of the furan ring is not predominant under the reaction conditions of this study, since the presence of acid sites seem to favor direct FFA rearrangement over ring hydrogenation.

It should be noted that increasing hydrogen pressure (from 7 to 43 bar) and correspondingly the total pressure (from 12 to 48 bar, respectively) not only increases the hydrogen concentration around the catalyst surface, but it also increases the liquid residence time as a consequence of the shrinking of the hydrogen bubbles. To eliminate the residence time effects and further elucidate on selectivity between FFA rearrangement vs. FFA hydrogenation (i. e., two sequential reactions after FFA formation and parallel between each other), the ratio of FFA rearrangement over deep hydrogenation products (i. e., S_rear/hyd_=Σ 4H2CP, CPEN, CPO, CPL yield/THFA yield) is used as proxy. Upon increasing the hydrogen pressure three‐fold, a non‐linear decrease in S_rear/hyd_ from 38 to 23 is observed, indicating higher preference for the furanic ring hydrogenation. Further increasing hydrogen pressure, first, four‐fold and then six‐fold, results in a minor S_rear/hyd_ decrease to approximately 20 for both cases. Thus, the increase of THFA yield with hydrogen pressure is not linear and does not lead to a significant reduction in the yield of the desired products (4H2CP and derivatives) even under the highest hydrogen pressures explored. Among the desired products, significant yields of 4H2CP are still present at 12 and 24 bar, indicating that the subsequent hydrogenation of intermediates remain a bottle‐neck towards production of CPO. Further increase of hydrogen pressure to 36 and 43 bar is beneficial but does not yet lead to full conversion of all intermediates (i. e., 4H2CP and CPEN) to CPO. Thus, increasing the hydrogen pressure leads to conditions conducive for CPO formation but does render entirely optimal results. Further increase of hydrogen pressure or different operating temperatures may still enhance the formation of THFA over that of the 4H2CP, CPO and CPEN conglomerate.

**Figure 5:**
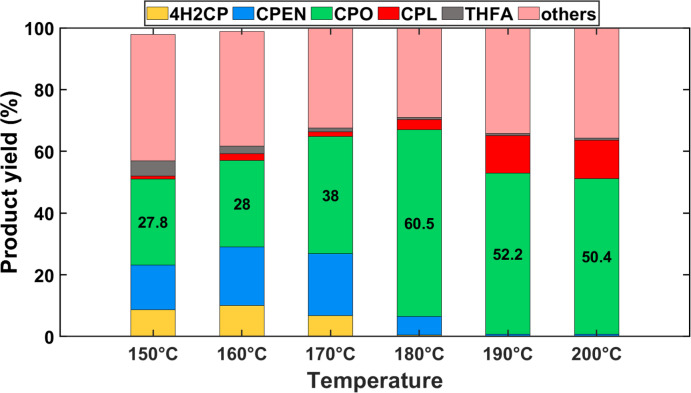
Effect of temperature on FFA hydrogenation to CPO using 1 wt.% FFA in water. Conditions: T=150–200 °C; P_total_=48 bar; W_cat_=0.2 grams of Cu/ZrO_2_ and β‐zeolite each; W_SiC_=3.6 grams; Q_feed_=0.2 mL min^−1^, $Q_{H_{2}}$ =4 N, WHSV=0.6 $g_{\mathrm{FFA}}\;g_{\mathrm{cat}}^{-1}\;h^{-1}$ . Figures within the green bar indicate CPO yield.

Figure 5 shows the effect of operating temperature on the product distribution for FFA hydrogenation over a mix of Cu/ZrO_2_ and β‐zeolite at a total pressure of 48 bar. Unlike previously discussed for the direct hydrogenation of furfural over supported‐Cu on acidic supports (Figure 3), increasing the temperature during the hydrogenation of FFA using Cu/ZrO_2_ and β‐zeolite does lead to significant CPO yield gains, even when performed under much greater WHSV (i. e., 0.06 vs 0.6, respectively). These findings are attributed to the presence of β‐zeolite, which catalyzes the rapid rearrangement of FFA rearrangement to 4H2CP, as opposed to the different acidic supports loaded with Cu (see Figure S11).

With respect to the hydrogenation activity (i. e., the direct hydrogenation of the FFA furan ring rendering THFA and that of 4H2CP to CPEN followed by CPO and CPL), all of these occur on the supported Cu catalysts. As shown in Figure 5, increasing temperature while keeping the total pressure constant decreases THFA yields from 5 to 0.5 % at 150 and 180 °C, respectively. Therefore, increasing the temperature favors the desired pathway over the furanic ring hydrogenation and FFA polymerisation reaction, thereby enhancing the atomic efficiency towards the desired rearrangement/hydrogenation pathway. This is attributed to a greater temperature dependence (Ea) for the FFA rearrangement over β‐zeolite than that that of furanic ring hydrogenation over Cu/ZrO_2_. Further increasing the temperature beyond 180 °C enables subsequent hydrogenation of CPO to CPL, accompanied with increase in formation of unaccounted products. Condensation of CPO or CPL with themselves to form bi‐cyclopentane or dimeric species can occur at these conditions. Such molecules might be responsible for the increase in ‘Others’ at higher temperatures.[^42^, ^43^]

### Furfural Hydrogenation to Cyclopentanone Over Cu Catalysts and β‐Zeolite Mixtures

Based on the observations so far, using an acidic catalyst such as β‐zeolite is key to catalyze FFA rearrangement and enable better atomic efficiency at lower contact times, thereby steering the selectivity towards 4H2CP and its hydrogenation products over parallel undesired reactions. Furthermore, combining acidic and Cu catalysts with higher hydrogen pressure and temperature was also essential to yield CPO when starting with FFA as substrate. It remains a question whether the same strategy will work using furfural as substrate. It should be remembered that the hydrogenation of furfural to FFA is a fast and selective step at relatively mild conditions (i. e., 150 °C and 7 bar H_2_ pressure), and this has been, so far, the only possible atomic efficient hydrogenation pathway starting from furfural.

**Figure 6:**
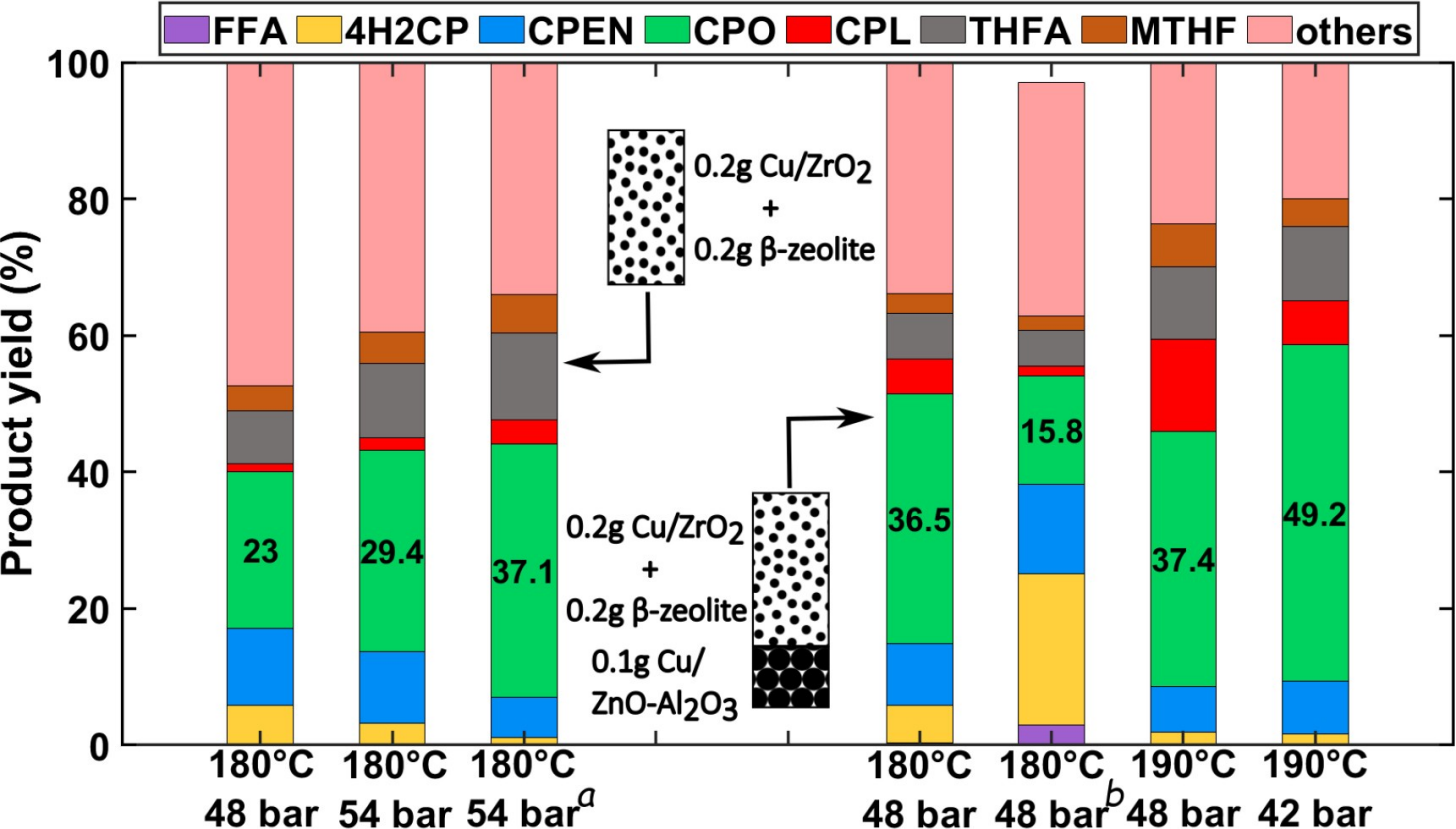
Furfural hydrogenation to CPO using 1 wt.% furfural in water. The three stacked bars on the left‐hand side represent mixed Cu/ZrO_2_ and β‐zeolite bed while those on right‐hand side represent commercial Cu/ZnO‐Al_2_O_3_ before the mixed bed. Conditions: Temperature and pressure are indicated on the X‐axis. W_cat_=indicated on the graph; Q_feed_=0.2 mL min^−1^, $Q_{H_{2}}$ =4 N, WHSV=0.06 and 0.4 $g_{\mathrm{furfural}}\;g_{\mathrm{cat}}^{-1}\;{\mathrm{hr}}^{-1}$ for mixed and sequential bed, respectively. (*a* :Q_feed_=0.15, $Q_{H_{2}}$ =3 NmL min^−1^, *b*: Q_feed_=0.4, $Q_{H_{2}}$ =8 NmL min^−1^). Figures in the green bar indicate CPO yield for respective temperature.

The left half of Figure 6 shows the results of furfural hydrogenation using a mixed bed consisting of Cu/ZrO_2_ and β‐zeolite as well as the optimized conditions (180 °C and 48 bar total pressure) earlier discussed for FFA hydrogenation. The first observation is that, indeed, this optimization strategy targeting the effective rearrangement of FFA to 4H2CP and its subsequent hydrogenation to CPO is also effective when starting with furfural. Significantly higher CPO yields and carbon balances are obtained than those in Figure 3 for different supports in similar temperature ranges. This confirms our hypothesis that FFA degradation is a major challenge in the conversion pathway and therefore, a catalytic strategy that enhances its selective rearrangement is key. Yet, substantial amounts of THFA and MTHF (products of deep ring hydrogenation indicated by grey and brown bars, respectively) are formed upon starting from furfural. Even greater yields of THFA and MTHF are observed upon increasing the total pressure to 54 bar. Hence, increasing pressure at higher operating temperature favors furanic ring hydrogenation of furfural, unlike observed when starting from FFA. As observed earlier, increasing H_2_ pressure (and thus liquid residence time) renders greater yields of FFA rearrangement and hydrogenation products, with a slight preference FFA hydrogenation (i. e., S_rear/hyd_ decreases slightly from ca. 3 to 2), and an increase in carbon balance. Additionally, increasing yields of MTHF with pressure suggest its formation pathway from THFA through dehydration/removal of hydroxyl group rather than 2‐methylfuran (2‐MF) hydrogenation as suggested elsewhere.[^44^, ^45^] Finally, while we concluded that furfural degradation was not a primary source of carbon losses in earlier experimental sets (with less acidic supports up to 160 °C), maximum 9 % of furfural degradation takes place in presence of β‐zeolite at 180 °C (see Table S2 in SI). The highest possible catalyst contacting (0.06) was used, while a contacting of 0.6 $g_{\mathrm{furfural}}\;g_{\mathrm{cat}}^{-1}\;{\mathrm{hr}}^{-1}$ was used in Figure 6. Therefore, upon decreasing the contacting with β‐zeolite as is the case for Figure 6, the extent of furfural degradation would be lower than 9 % and can be neglected.

While combining β‐zeolite with Cu catalysts under the conditions that favor FFA rearrangement and subsequent hydrogenation, respectively, remains an attractive strategy for one‐pot furfural hydrogenation, only ca. 40 % furfural‐to‐CPO (or ca. 60 % furfural‐to‐4H2CP derivatives) yield is attained. Alternatively, using a two‐step strategy starting with furfural hydrogenation to FFA at milder conditions (e. g., 150 °C and 12 bar with Cu/ZnO‐Al_2_O_3_) followed by FFA hydrogenation to CPO in a more severe environment (180 °C and 48 bar, combining β‐zeolite and Cu‐catalysis), renders more attractive yields (i. e., 60 % furfural‐to‐CPO or ca. 70 % furfural‐to‐4H2CP derivatives). This raises the question whether a staged reactor could offer the benefits of a having single reactor (i. e., lower CAPEX) with the possibilities to optimize individual reactions separately, to a certain extent. Using such a staged reactor system is practised in industry (e. g., employing a guard bed reactor followed by staged hydrogen inlets in a hydro‐treating plant within a refinery^[46]^). Yet, using uniform process condition might have benefits of potentially lower CAPEX and ease of operation. Therefore, we investigated a sequential bed strategy containing, first, a commercial Cu/ZnO‐Al_2_O_3_ bed for furfural to FFA, and then, the mixed Cu/ZrO_2_ and β‐zeolite catalytic bed for performing FFA rearrangement/hydrogenation to CPO.

The right‐hand side of Figure 6 shows the results of furfural hydrogenation in the staged reactor. The absence of β‐zeolite in the first segment of the reactor presumably limits the furfural condensation pathway, while its presence in the second segment catalyzes the desired rearrangement of FFA, thereby leading to greater carbon balances. The improvement in carbon balance upon using sequential bed vs. mixed bed for 180 °C and 48 bar total pressure is noticeable. However, we may argue that these experiments are not directly comparable, since the sequential bed has higher catalyst loading (both Cu and acid sites) than the mixed bed. Thus, greater yields of rearrangement and further hydrogenated products may be attributed to that. On the other hand, the yield to products such as THFA and MTHF (2‐methyltetrahydrofuran) is similar to that with mixed bed. Therefore, THFA and MTHF indeed originate from furan ring hydrogenation of furfural rather than FFA irrespective of the nature of supports used. Presumably, furan ring hydrogenation takes place already on the Cu/ZnO‐Al_2_O_3_ bed, in parallel to the very rapid hydrogenation of furfural to FFA. Still, the synthesis of FFA and subsequent rearrangement/hydrogenation remains the primary pathway, as revealed by the product distribution, which is very similar to that of direct FFA hydrogenation. The selectivity to the rearrangement products over that of deep hydrogenation products (S_rear/hyd_) is greater than that in the mixed bed configuration (around 6.5. vs 3, respectively). Thus, the staged reactor strategy is indeed an attractive strategy for one‐pot conversion of furfural to CPO. Further optimization of residence time, pressure and temperature can lead to better yields. Exploration with shorter contact times (i. e., fifth bar from left in Figure 6) reveal the expected product distribution, consisting of a relatively large fraction of intermediate products (i. e., traces (<5%) of unconverted FFA, and significant large yields of 4H2CP and CPEN), and comparatively lower yields of final products like CPO, CPL and THFA and MTHF. Remarkably, the yield to CPL (from CPO hydrogenation) is very low in all experiments up to 180 °C. Presumably, that reaction step imposes important energy barriers. Further increasing temperature to 190 °C, 48 bar and the reference contact time (i. e., sixth bar from left in Figure 6) show visible improvements in yield to all quantified products, including those from FFA rearrangement and hydrogenation (i. e., desired products), and thus a better carbon balance, albeit a substantial increase in the extent of hydrogenation of furanic ring to THFA and MTHF. The S_rear/hyd_ decreases from ca. 6.2 to 3.3 when raising temperature from from 180 to 190 °C. In addition, CPO hydrogenation to CPL spikes at 190 °C, which may be unwanted when targeting CPO as final product. This re‐iterates the necessity to decouple the hydrogenation scheme into two steps for maximum CPO yields irrespective of the nature of catalysts or supports used. Additionally, such a system could provide an added flexibility of selecting the end product based on market trends, making it an attractive proposition based on its modularity.

### Long‐Term Catalyst Stability Test

**Figure 7:**
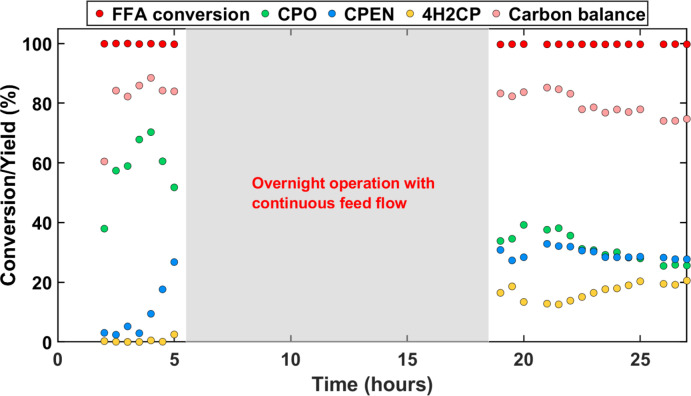
FFA hydrogenation to CPO using 1 wt.% FFA in water. Conditions: T=180 °C; P_total_=48 bar; W_cat_=0.2 grams of Cu/ZrO_2_ and β‐zeolite each; W_SiC_=3.6 grams; Q_feed_=0.2 mL min^−1^, $Q_{H_{2}}$ =4 NmL min^−1^, WHSV=0.6 $g_{\mathrm{FFA}}\;g_{\mathrm{cat}}^{-1}\;h^{-1}$ .

Evaluating long term catalyst activity is key for future process and catalyst development. While numerous works show stability of Cu catalysts used by consecutive regeneration and activity testing, contradicting evidences of non‐noble metals such as Cu and Ni leaching out under hydrothermal conditions are prevalent too.[^47^, ^48^, ^49^] In consideration of these evidences, reaction conditions for FFA hydrogenation to CPO were chosen as being the more severe of the two steps. As seen from Figure 7, there is a significant drop in CPO yields from 60 to 70 % in the initial 3 hours to 30 % at the end of 30 hours of time on stream. Decrease in yields of hydrogenated product coinciding with increasing 4H2CP yield indicates that the catalyst is unable to hydrogenate the intermediates resulting from FFA rearrangement. The filtrate of the samples showed presence of Cu (~10 ppm) by ICP‐OES (detection limit 10 ppb) indicating leaching to be one of the causes of deactivation; supported by leaching of Cu from CuZnAl catalysts for furfural hydrogenation to CPO^[50]^ at 150 °C. This results in approximately 4 mg of Cu lost within 30 hours of continuous operation in this work.

**Figure 8:**
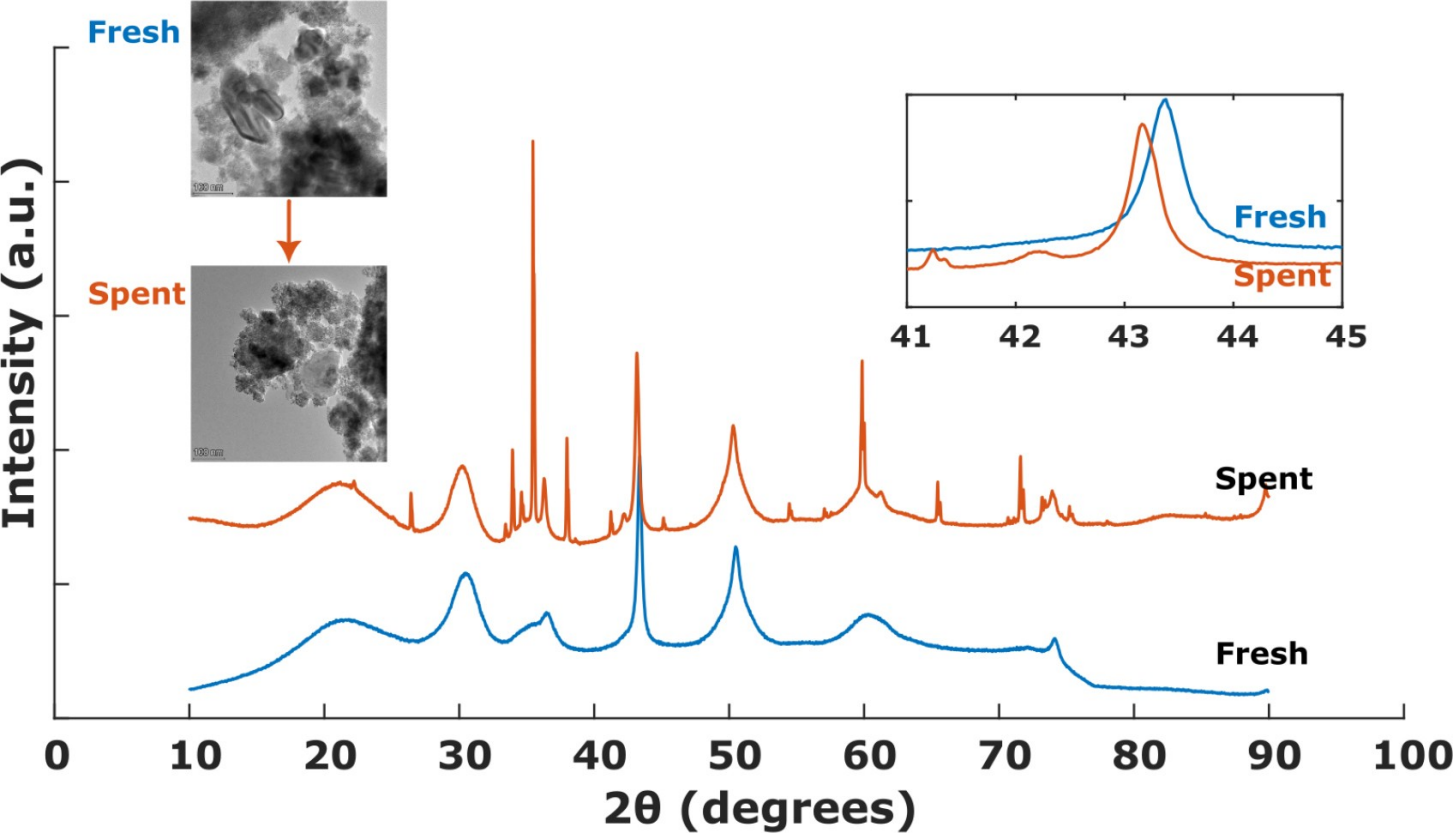
XRD pattern of fresh vs. spent catalyst. Inset on the top right‐hand side shows an enlarged segment of XRD pattern in Cu^0^(111) region while the TEM images of fresh and spent catalysts are shown on the top left‐hand side.

While Cu leaching is one of the contributing factors for loss in activity, the formation of polymeric deposits on the catalyst surface along with other factors, such as metal sintering or loss of crystalline structure cannot be ruled out. It should be noted that all of the experiments carried out in this work showed yellow coloration of the liquid sample at the reactor outlet as compared to the initial colorless feedstock used (see Figure S13 in SI). Upon letting the liquid samples stay undisturbed for at least a week, these yellow colored samples resulted in formation of brown precipitates (solids) at the bottom, suggesting the formation of condensates that are in line with the incomplete carbon balances. In addition, XRD patterns and TEM analysis of the fresh and spent catalysts were compared (see Figure 8). While the Cu particle sizes do not show significant sintering, a shift in Cu^0^(111) reflection using XRD can be observed after exposure to catalytic reactions. Additionally, a decrease in FWHM of Cu(111) hints towards an increase in crystallite size, and the increasing long‐order crystallinity of ZrO_2_ from its originally amorphous structure suggests induced strain on the crystal lattice while operating at reaction conditions. The absence of ZrO_2_ crystalline peaks in the freshly prepared catalyst can be assumed either due to the support′s amorphous nature, or a very fine crystal size. Regardless, intense reflections for the spent catalyst at 2*θ* of about 30.2°, 35.2° and 60.2° can be ascribed to (011), (110) and (121) planes of tetragonal ZrO_2_, respectively. While this work performed post‐mortem analysis of morphological properties of the catalyst upon end of operation, a systematic study of such phenomena with time‐on‐stream operation might be insightful.

## Conclusions

This work focuses on furfural hydrogenation to CPO in a flow reactor using copper catalysts with different acidic supports (i. e., ZrO_2_, ZnO−Al_2_O_3_ and MgO−Al_2_O_3_) at 150–200 °C and 7–43 bar H_2_ pressure. Mechanistically, our results evidence that furfural rapidly hydrogenates to FFA, which subsequently undergoes rearrangement to 4H2CP and further hydrogenation to CPEN, CPO and CPL. FFA rearrangement appears to be a major bottle‐neck in synthesis of CPO due to a parallel polymeric reaction pathway. The rearrangement is selectively catalyzed in presence of weak acid sites. Therefore, β‐zeolite was added to the bed in combination with the supported‐Cu catalysts to enhance CPO yields. This combination was utilized in three different bed configurations: 1) a single step using a homogeneous bed; 2) a single step using two‐staged catalytic bed; and 3) two catalytic beds. The final approach combined with different operating conditions for a staged conversion of furfural, first to FFA and then to CPO, results in highest CPO yields, i.e, 60 %. The first two choices result in 23 and 37 % CPO yields using similar conditions (180 °C and 38 bar H_2_), mainly due to parallel ring hydrogenation of furfural. Long‐term activity testing shows an irreversible deactivation upon 30 hours of operation due to Cu leaching. Future works could focus on development of leaching‐resistant heterogeneous catalyst rich in weak acid sites. Besides, a two‐step production strategy provides extra modularity and versatility in the value chain, which is advantageous to promote the use of renewable sources like biomass for chemical production.

## Experimental

### Catalyst Synthesis

Cu/ZrO_2_ catalysts were synthesised as stated elsewhere.^[24]^ Typically, 0.01 mol of both Cu precursor (Cu(NO_3_)_2_. 2.5H_2_O) and zirconia precursor (ZrO(NO_3_)_2_) were dissolved in 80 mL of deionised water to form salt solution A. Solution B consisted of 0.2 mol NaBH_4_ in 80 mL deionised water. These solutions were then added to a colloid mill and mixed rapidly at 10000 rpm for 3 minutes. The resulting solid suspension was then transferred to a Teflon‐lined autoclave and aged at 150 °C in an oven for 48 hrs. The obtained precipitate was then washed and centrifuged until pH of the filtrate reached 7. This solid was then dried at 90 °C overnight and calcined in air at 500 °C for 6 hrs. The Cu catalysts were pre‐reduced in a tubular oven at 300 °C for 3 hours with 20 % H_2_/80 % N_2_ mixture, passivated with 5 % O_2_/Ar mixture.

The procedure to synthesise Cu/MgO‐Al_2_O_3_ remained the same except for 0.016 mol of Cu precursor, 0.016 mol of alumina precursor (Al(NO_3_)_3_) and 0.032 mol of magnesia precursor (Mg(NO_3_)_2_). This was done to ensure the same loading of Cu (approximately 30 wt.%) across both the catalyst supports synthesized/used.

β‐zeolite (Si : Al ‐ 25) was purchased from Alfa Aesar in its ${\mathrm{NH}}_{4}^{+}$ form and calcined at 300 °C for 2 hours to obtain H^+^ form.

### Catalyst Characterisation

X‐ray powder diffraction (XRD) patterns were recorded on a Rigaku MiniFlex 600 diffractometer equipped with a Cu tube, Kβ (x2) filter. Signal was measured in the range 10–90° 2*θ* with a step size of 0.02° and counting time of 2 seconds per step.

Next, the textural analysis of the specimens was conducted by low temperature (88 K) N_2_‐physisorption using Micromeritics TriStar II 3020.The specific surface area was determined by Brunauer‐Emmet‐Teller (BET) equation,^[51]^ while the total pore volume (V_tot_) was obtained at the relative pressure P/P_0_=0.99 using BJH method.^[52]^ The same method was applied for the calculation of the average pore diameter (D_pores_) and pore size distribution.

Catalyst acidity and reducibility were investigated by NH_3_ TPD and H_2_ TPR, respectively. Both were performed with Micromeritics Autochem II instrument. Typically, 100 mg of the as synthesized catalysts were loaded into a U‐tube quartz glass reactor. For NH_3_ TPD experiments, the catalysts were reduced in‐situ with 10/90 H_2_/Ar mixture (vol.:vol.) at 300 °C for 2 hours at a ramp rate of 2 °C/min. The catalyst bed was then cooled down to 90 °C flushed with Ar for 60 minutes. Then 2/98 NH_3_/Ar mixture (vol.:vol.) was passed for 60 minutes followed by Ar purging. Ar was flowed continuously with a ramp rate of 10 °C/min. For H_2_ TPR, the loaded catalysts were first degassed under Ar flow at 200 °C for 2 hours. The catalyst bed was then cooled down to 20 °C. A mixture of 10/90 H_2_/Ar mixture (vol.:vol.) was flowed through the catalyst bed with a ramp rate of 10 °C/min passing through a cold trap was prepared using liquid N_2_ – isopropanol slurry.

Finally, the Cu surface area was determined by 2‐mercaptobenzimidiazole (MBI) titration. Cu catalysts were reduced in a tubular oven at 300 °C for 3 hours with 20/80 H_2_/N_2_ (vol.:vol.) at 2 °C/min followed by passivation with 5/95 O_2_/Ar mixture. Typically, 10 mg of catalysts were kept stirring in 20 mL 100 μM solution of 2‐mercaptobenzimidiazole (MBI) in distilled water overnight. The solution was then filtered and analyzed with a UV‐vis spectrometer for decrease in the intensity at 300 nm. Cu surface area was then determined using MBI packing density as mentioned elsewhere.^[27]^

### Activity Testing

The required mass of pre‐reduced and passivated catalyst was diluted with SiC and packed in a stainless steel tubular reactor. A 10 micron and 2 micron frits with metal gaskets were placed upstream and downstream the catalytic bed to prevent catalyst fouling and washing away of the catalyst bed, respectively. The reactor was then leak tested at 1.25 times the operating pressure with nitrogen prior to flow experiments. Hydrogenation of furfural/furfuryl alcohol was performed in an upflow packed bed reactor placed in an oven with a gas‐liquid separator at the reactor outlet from where liquid samples were taken. The liquid was pumped using an ISCO pump while the reactor pressure was maintained using a back‐pressure controller. In a typical experiment, the substrate solution was flowed through the reactor after a successful leak test to vent out the gas and to ensure no channeling through the packing. After the liquid reached the sampling point, the gas flow was turned on followed by oven heating. As the desired temperature was reached, reaction start time was recorded. Reproducibility tests with catalysts synthesized from different batches were performed and can be found in Figure S15 in SI.

### Product Analysis

Samples collected from the reactor outlet were analysed by gas chromatography (Shimadzu GC) using a capillary column CPSiL 5CB (30 m (length) * 0.52 mm (i.d.)) * 1 μm connected to a flame ionization detector. The performance was studied in terms of (%) conversion of the substrate (furfural or furfuryl alcohol) and (%) product yield as defined below

(1) $$\mathrm{Conversion}\;\left(X_{i}\right)=\frac{C_{\mathrm{substrate},\;\mathrm{initial}}{-C}_{\mathrm{substrate},\;\mathrm{reactoroutlet}}}{C_{\mathrm{substrate},\;\mathrm{initial}}}\cdot 100$$

(2) $${\mathrm{Yield}}_{i}\left(Y_{i}\right)=\frac{C_{\mathrm{product},\;\mathrm{reactoroutlet}}}{C_{\mathrm{substrate},\;\mathrm{initial}}}\cdot 100$$

(3) $${\mathrm{Selectivity}}_{i}\left(S_{i}\right)=\frac{C_{\mathrm{product},\;\mathrm{reactoroutlet}}}{C_{\mathrm{substrate},\;\mathrm{initial}}{-C}_{\mathrm{substrate},\;\mathrm{reactoroutlet}}}\cdot 100$$

(4) $$\mathrm{Others}=\mathrm{Conversion}-{\Sigma (\mathrm{Yield}}_{i})$$

Products quantified for equations (2), (3) and (4) are FFA, 4H2CP, CPEN, CPO, CPL, 2‐MF, MTHF, THFA and LA.

(5) $$\mathrm{Liquid}\;\mathrm{residence}\;\mathrm{time}\;\left(\tau \right)=\frac{V_{R}\cdot \epsilon_{\mathrm{liquid}}}{Q_{\mathrm{liquid}}}$$

## Conflict of Interests

The authors have no conflict of interest to declare.

1

## Supporting information

As a service to our authors and readers, this journal provides supporting information supplied by the authors. Such materials are peer reviewed and may be re‐organized for online delivery, but are not copy‐edited or typeset. Technical support issues arising from supporting information (other than missing files) should be addressed to the authors.

Supporting Information

Supporting Information

## Data Availability

The data that support the findings of this study are available from the corresponding author upon reasonable request.
