# Supplementary material for: Intensifying Cyclopentanone Synthesis from Furfural Using Supported Copper Catalysts
Source: ChemSusChem. 2024 Nov 8;18(4):e202401484. doi: 10.1002/cssc.202401484 (PMC11826132; doi:10.1002/cssc.202401484)
Supplement: Supplementary file 1 — Supporting Information [file CSSC-18-e202401484-s001.pdf]

ChemSusChem

Supporting Information

Intensifying Cyclopentanone Synthesis from Furfural Using Supported Copper Catalysts

Adarsh Patil, Maurik Engelbert van Bevervoorde, and Fernanda Neira d'Angelo*

Intensifying cyclopentanone synthesis from furfural using supported copper catalysts

Adarsh Patil,[a] Maurik Engelbert van Bevervoorde,[a] Dr. Fernanda Neira d'Angelo*[a]

[a] Sustainable Process Engineering Group, Eindhoven University of Technology,
P.O. Box 513, 5600 MB, Eindhoven, The Netherlands
E-mail: m.f.neira.dangelo@tue.nl

Supplementary Information

Contents

1. Catalyst characterisation **1**
1.1. XRD pattern of calcined and reduced catalysts . . . 1
1.2. SEM-EDX results . . . 1
1.3. N_2 physisorption . . . 3
1.4. Cu surface area using MBI . . . 4
1.5. H_2-TPR of supported-Cu catalysts . . . 4
1.6. NH_3-TPD of H-ZSM5 and Ga-ZSM5 catalysts . . . 5

2. Furfural hydrogenation over supported-Cu catalysts **5**

3. Furfuryl alcohol rearrangement over acidic catalysts **6**
3.1. Catalyst screening for FFA rearrangement to 4H2CP . . . 6
3.2. Effect of FFA concentration on 4H2CP yield . . . 6

4. Assigning carbon loss in furfural hydrogenation to CPO **7**
4.1. Furfural degradation over solid catalysts . . . 7
4.2. Polymeric species from FFA - visualization and structures . . . 7
4.3. Solvent screening and protecting species for hydroxyl group on FFA . . . 8

5. Reproducibility test with different catalyst batches of synthesized Cu/ZrO_2 **9**

1. Catalyst characterisation

1.1. XRD pattern of calcined and reduced catalysts

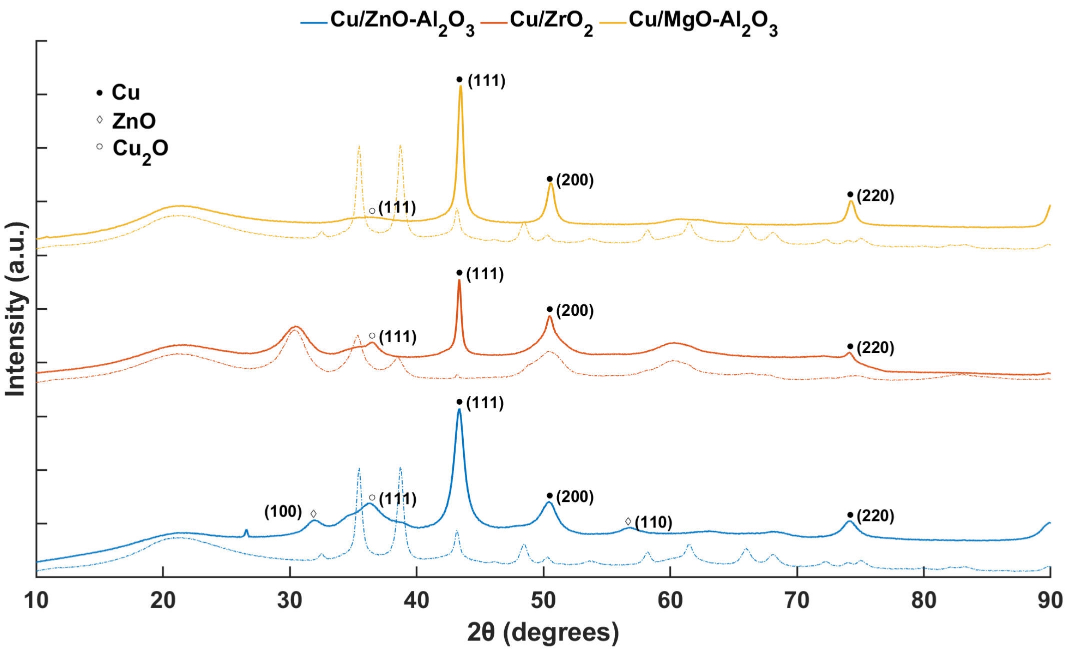

Figure S1. XRD patterns of the copper catalysts used. Dashed and solid lines indicate calcined and reduced forms, respectively. Figures within parenthesis next to the markers indicate the crystal plane of the respective markers indicated in the top left corner of the image.

1.2. SEM-EDX results

Table S1. Average measured atomic and weight concentrations of used catalysts with 95% confidence interval

Catalyst	Element	Atomic concentration (%)	Weight concentration (%)
Cu/ZnO-Al_2O_3	Cu	34.79 ± 1.57	54.08 ± 1.66
	Zn	14.45 ± 0.12	23.11 ± 0.15
	Al	10.85 ± 1.93	7.18 ± 1.38
	O	39.92 ± 0.24	15.63 ± 0.14
Cu/ZrO_2	Cu	18.29 ± 2.91	29.36 ± 1.84
	Zr	19.62 ± 3.24	45.21 ± 3.12
	O	62.10 ± 6.15	25.43 ± 4.95
Cu/MgO-Al_2O_3	Cu	8.24 ± 0.14	24.00 ± 0.30
	Mg	20.03 ± 0.34	21.70 ± 0.31
	Al	6.68 ± 0.02	8.10 ± 0.10
	O	64.7 ± 0.59	46.15 ± 0.65

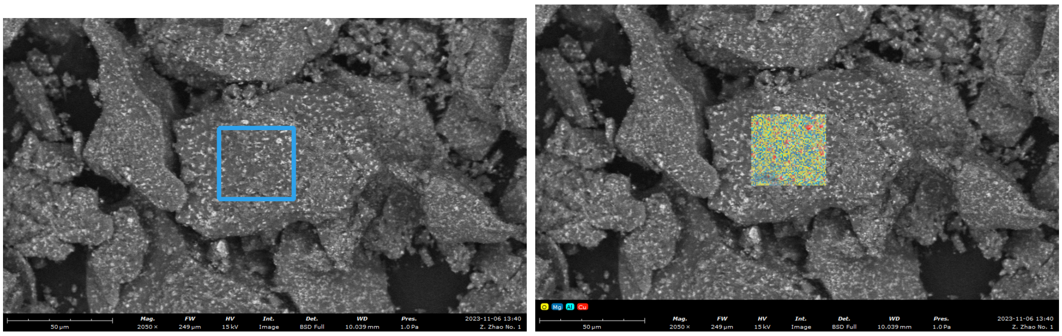

Figure S2. SEM-EDX surface mapping of Cu/MgO-Al_2O_3

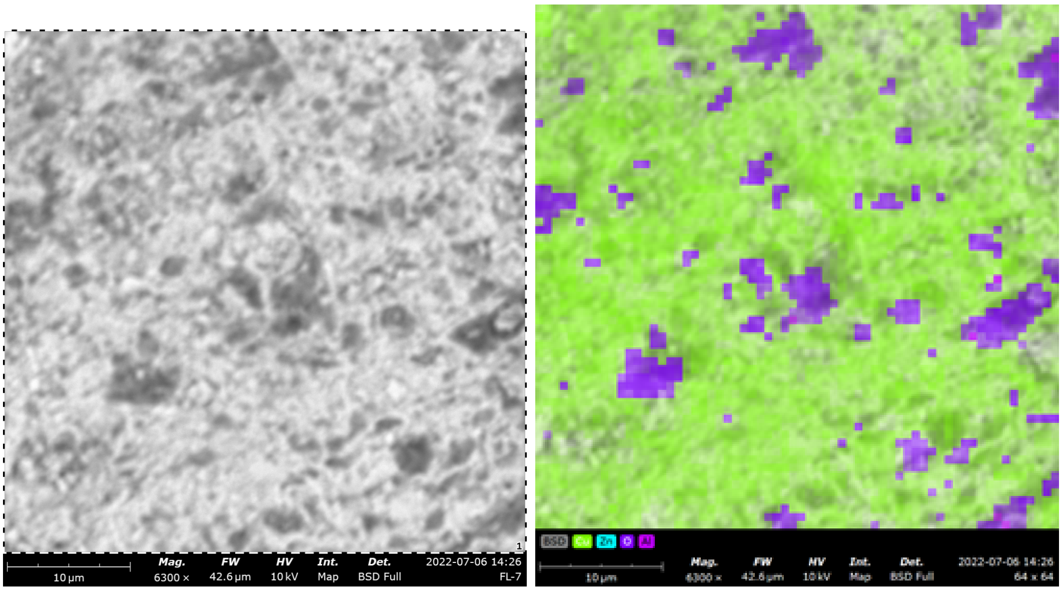

Figure S3. SEM-EDX surface mapping of Cu/Zn-Al_2O_3

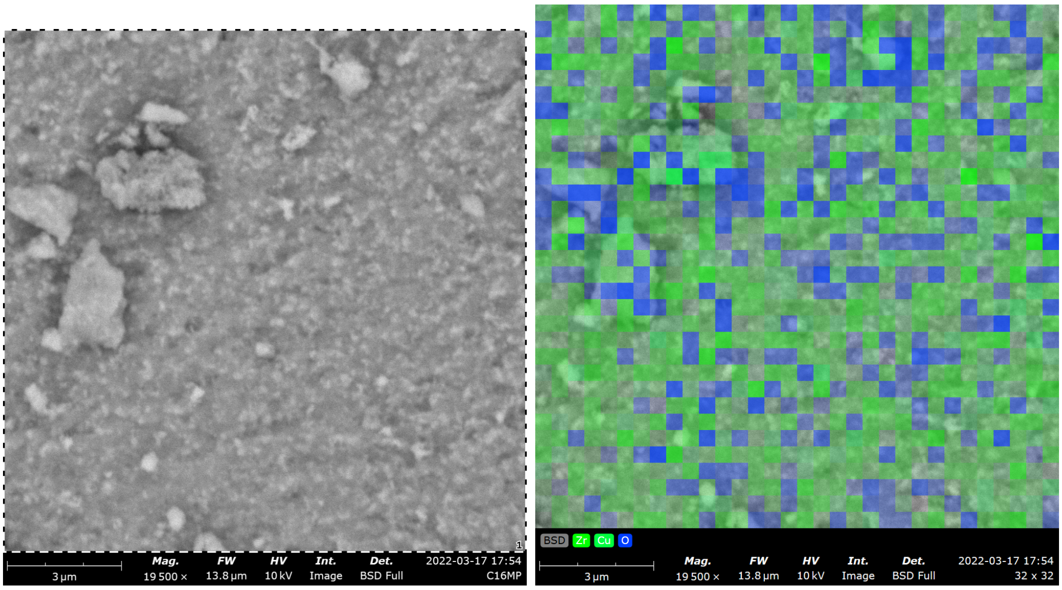

Figure S4. SEM-EDX surface mapping of Cu/ZrO_2

1.3. N_2 physisorption

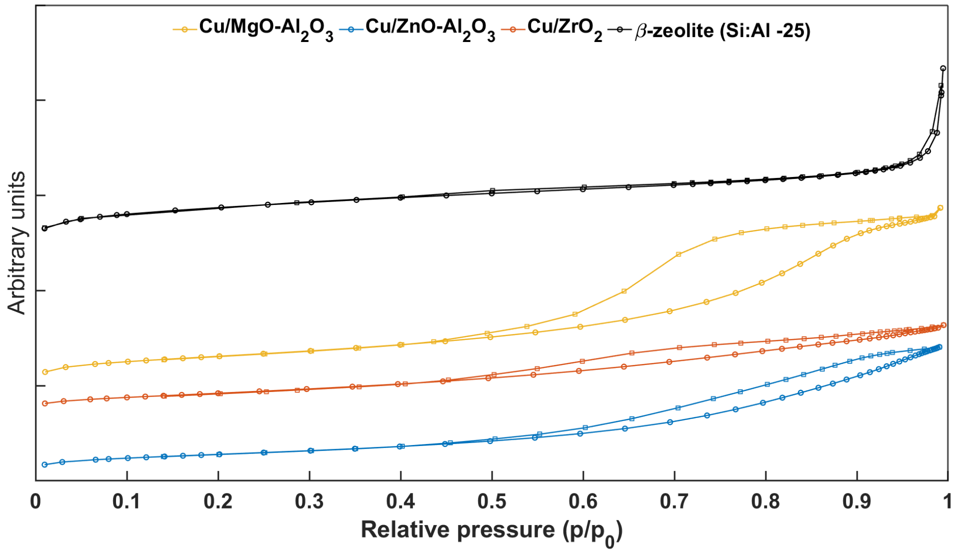

Figure S5. N_2 physisorption isotherms for the catalysts. BET results obtained were after calcination and pre-reduction. The 'circle' markers indicate adsorption curve while the 'square' markers indicate desorption curve.

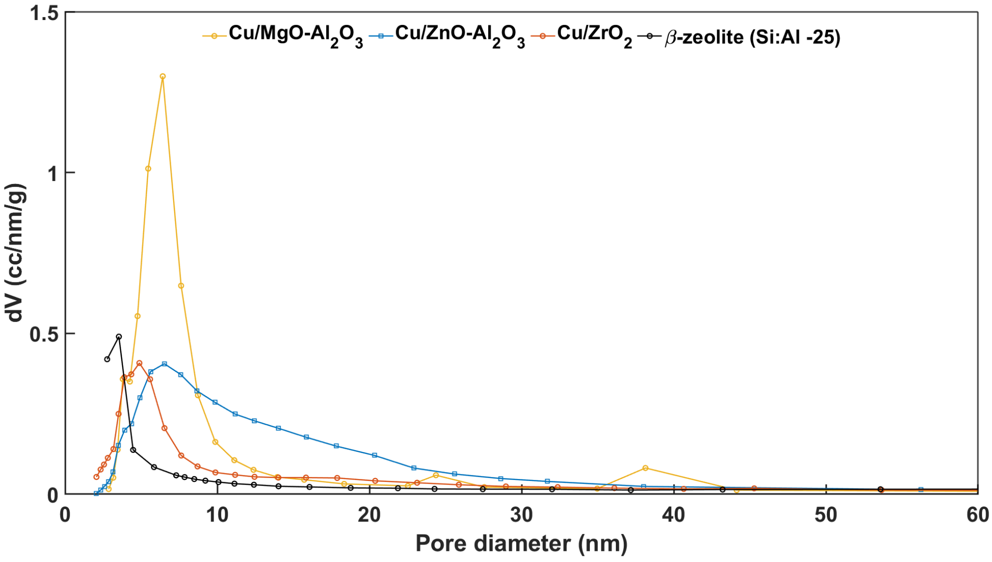

Figure S6. Pore size distribution curve for the catalysts using desorption branch of the isotherm.

1.4. Cu surface area using MBI

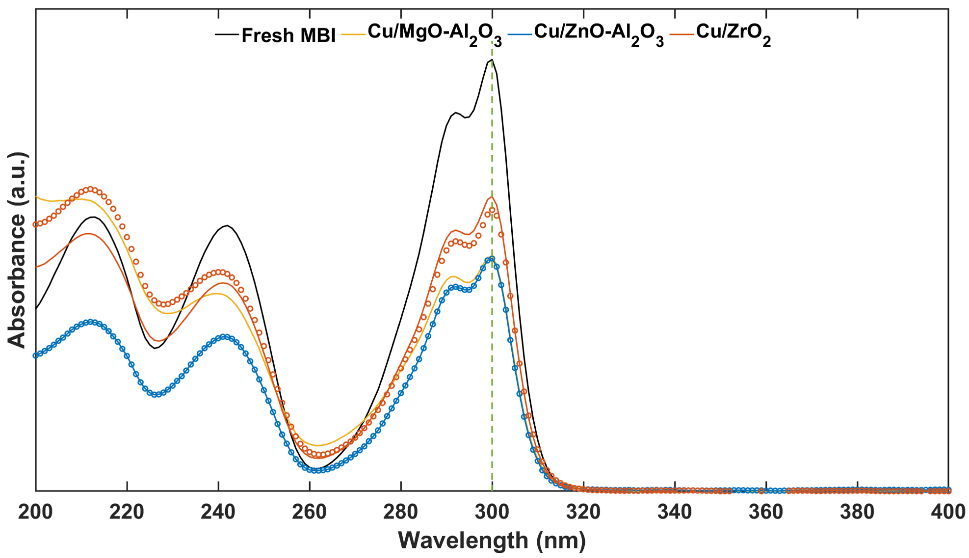

Figure S7. UV-vis spectrum of 100 μM MBI solution with and without suspended Cu catalysts overnight. Markers denote duplicate experiments for the same supported catalysts. Green dashed line represents the characteristic wavelength for determination of MBI concentration in the solution.

1.5. H_2-TPR of supported-Cu catalysts

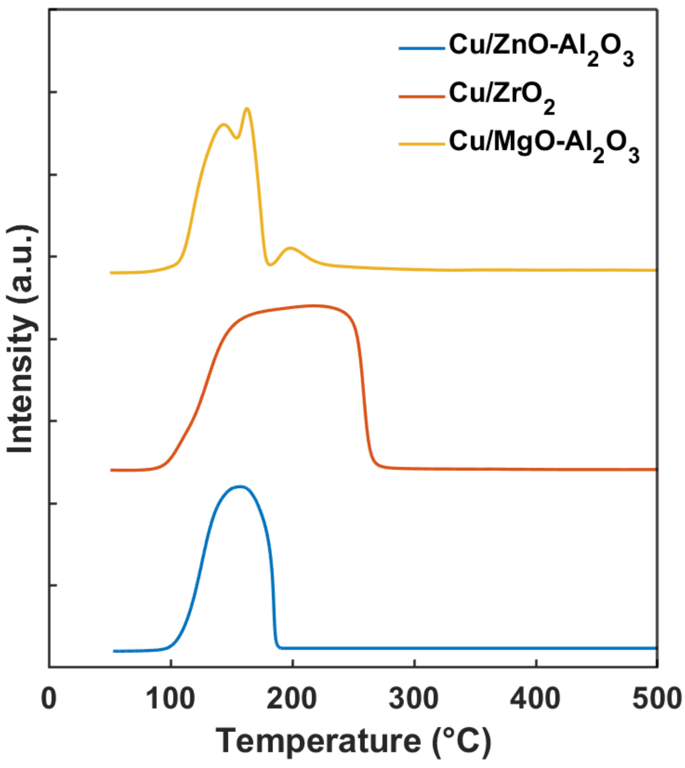

Figure S8. H_2-TPR of supported-Cu catalysts

1.6. NH_3-TPD of H-ZSM5 and Ga-ZSM5 catalysts

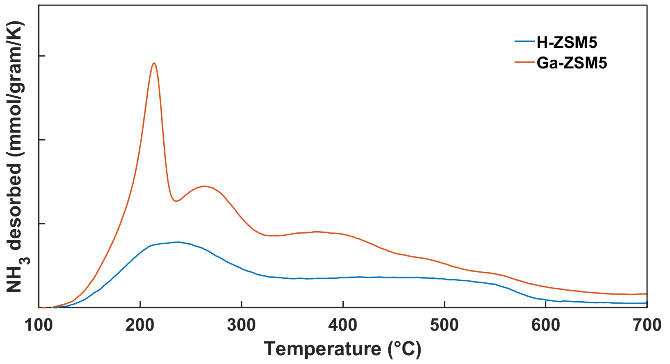

Figure S9. NH_3-TPD of H-ZSM5 and Ga-ZSM5. Ga-ZSM5 was prepared by ion-exchange of H-ZSM5 with 0.1 M $Ga(NO_3)_3$ aqueous solution at 80°C over 1 hour. Subsequent drying and calcination of Ga-ZSM5 was done at 80 and 300°C, respectively.

2. Furfural hydrogenation over supported-Cu catalysts

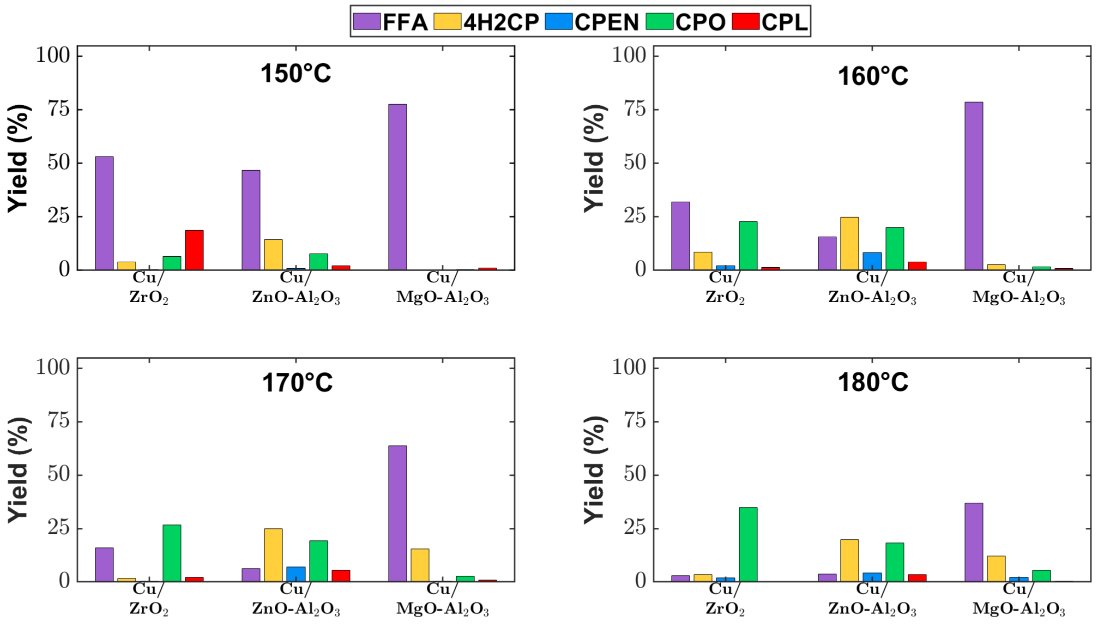

Figure S10. Comparison of different supports on furfural hydrogenation to cyclopentanone using 1 wt% furfural in water. Conditions: T = 150 - 180 °C; P_{total} = 12 bar; W_{cat} = 1 gram; W_{SiC} = 3 gram; Q_{feed} = 0.1 $mL\,min^{-1}$, Q_{H_2}= 5 $NmL\,min^{-1}$; and WHSV = 0.06 $g_{furfural}\,g_{cat}\,hr^{-1}$

3. Furfuryl alcohol rearrangement over acidic catalysts

3.1. Catalyst screening for FFA rearrangement to 4H2CP

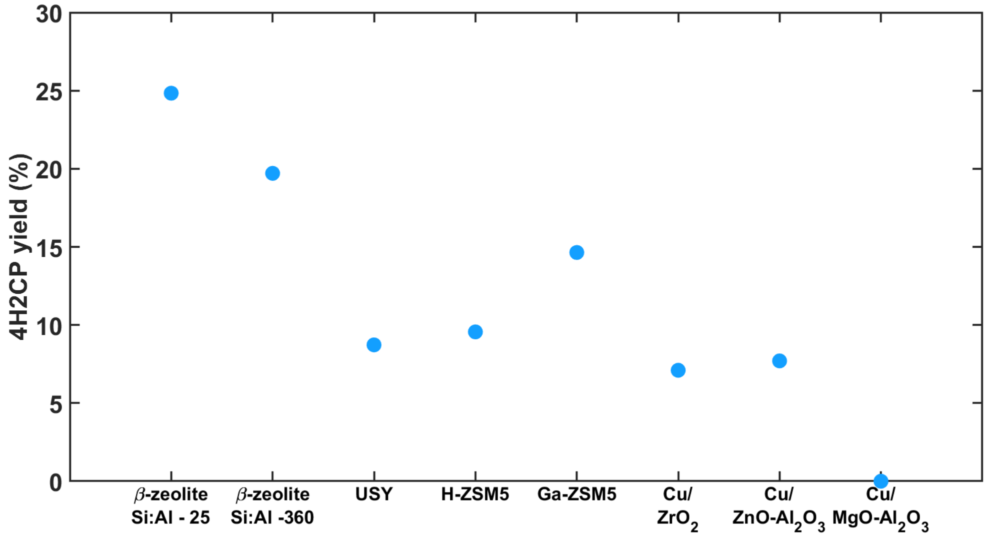

Figure S11. 4H2CP yilds obtained with different catalysts using FFA. Conditions: T = 150°C, FFA:Catalyst = 10:1 (w:w), Reaction time = 8 minutes.

3.2. Effect of FFA concentration on 4H2CP yield

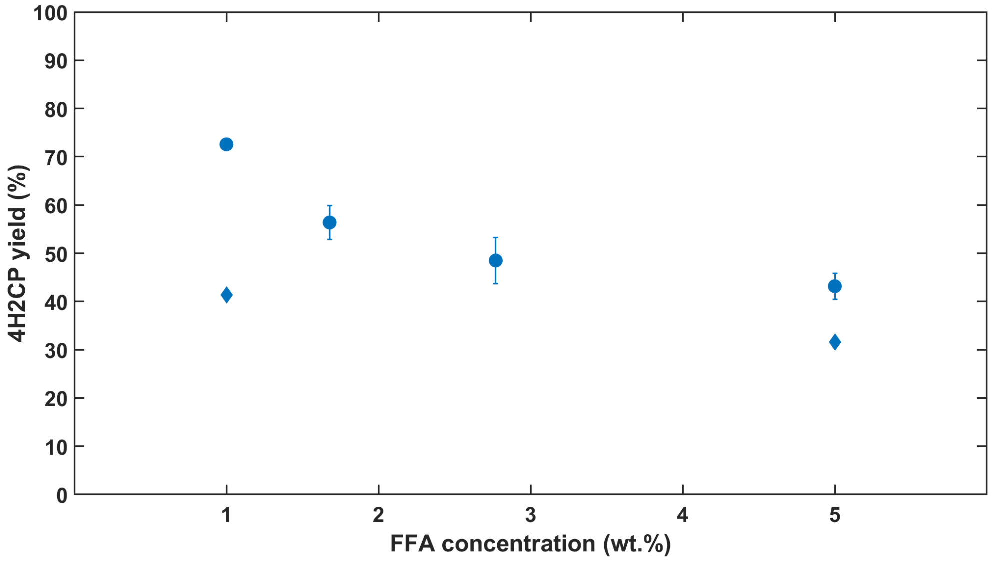

Figure S12. 4H2CP yield for different initial FFA concentrations in water. Conditions: T - 150°C; Reaction time - 30 minutes; FFA:catalyst - 10:1 (w:w); WHSV = 0.6 $g_{FFA}\ g_{cat}^{-1}\ hr^{-1}$. Circles and diamond indicate β-zeolite and TiO_2 respectively. **Note** - All concentrations for β-zeolite reached complete conversion and yield can be interpreted as selectivity. 65 and 100% FFA conversion were achieved with TiO_2 for 1 and 5 wt.% respectively.

4. Assigning carbon loss in furfural hydrogenation to CPO

4.1. Furfural degradation over solid catalysts

Table S2. Furfural degradation with different solid acid catalysts with 1 wt.% furfural in water.

Entry	Catalyst	WHSV ($g_{furfural}\, g_{cat}^{-1}\, hr^{-1}$)	Temperature (°C)	$X_{furfural}$ (%)
1	Blank[a]	0.06	160	2.1
2	ZrO_2	0.06	150	2.1
3		0.06	160	2.8
4	β-zeolite (Si:Al - 25)	0.06	170	4.4
5		0.06	180	9.4
6	Cu/ZnO-Al_2O_3	0.06	170	0.4
7		0.06	180	1.1

[a] No catalyst, only inert packing (4 grams SiC)

4.2. Polymeric species from FFA - visualization and structures

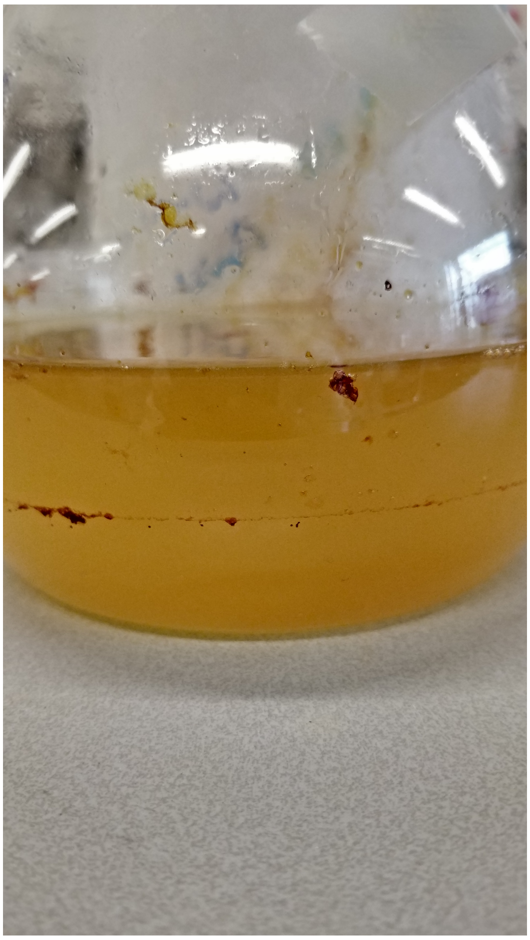

Figure S13. Visual depiction of the reaction sample obtained during the long-term FFA hydrogenation to CPO.

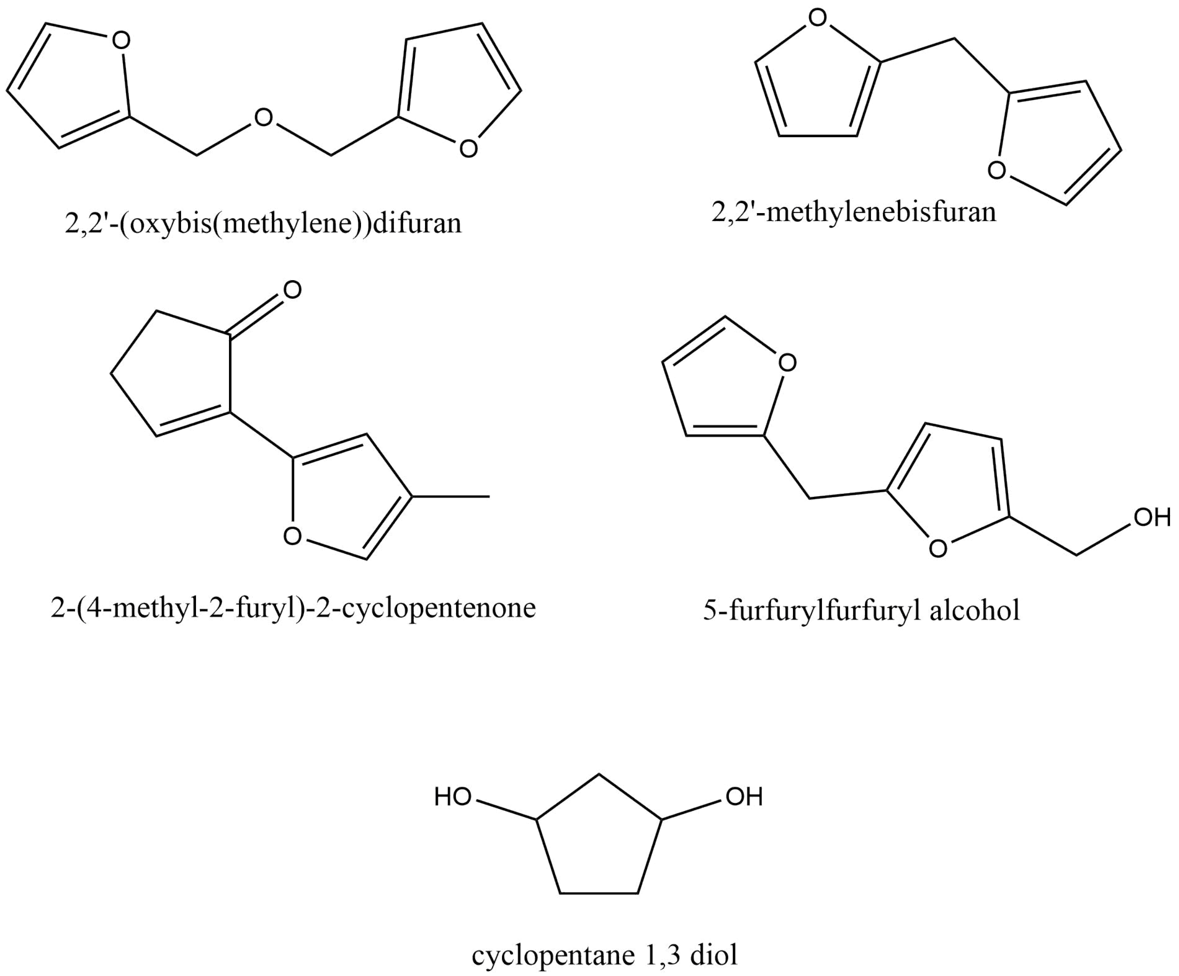

Figure S14. Molecules detected using GC-MS for FFA hydrogenation to CPO. **NOTE** : Products resulting from CPO self-condensation were detected in trace amounts.

4.3. Solvent screening and protecting species for hydroxyl group on FFA

Table S3. Effect of co-solvent on conversion of 1 wt.% FFA in 10 mL aqueous/organic solution at 150°C over β-zeolite

Entry	Solvent	X_{FFA} (%)	Y_{4H2CP} (%)	Carbon balance (%)
1	50 wt.% 1,4-dioxane	78.6	28.1	50.3
2	50 wt.% dimethylsulfoxide	99.3	5.8	6.7
3	50 wt.% dimethylformamide	6.1	0.6	94.4
4	25 wt.% tetrahydrofuran[a]	97.4	29.6	33.0
5	Hexamethyldisilazane[b]	22.3	0.1	77.9
6	(3-Aminopropyl)trimethoxysilane[b]	0.1	0	>99

[a] using flow reactor at 12 bar [b] equimolar amounts of molecule : FFA

1,4-dioxane and dimethylsulfoxide (DMSO) are selected based on literature [12,53] and dimethylformamide (DMF) due to its similarity to dimethylsulfoxide. Tetrahydrofuran (THF) was selected due to its ability to enhance selectivity of similar reactions, e.g. furfural and 5-hydroxymethylfurfural production [54]. Hexamethyldisilizane and (3-aminopropyl)trimethoxysilane (APTMS) were chosen due to their use as protecting agents [55,56]. In particular, these groups can protect FFA from undergoing polymerisation. Table S3 shows that these silane compounds, and especially APTMS are succesful in preventing FFA polymerisation, and almost no carbon loss. Absence of 4H2CP indicates that FFA rearrangement is initiated via its hydroxyl group. This corroborates the mechanism proposed by Mironenko et al., where the first step in FFA rearrangement is protonation of the alcohol group, which is blocked by steric hindrance by APTMS protection. [37] However, this protection of hydroxyl group renders no 4H2CP formation. Other solvents either prevent FFA from reacting (i.e., DMF) or exacerbate loss of carbon balance via polymerisation (DMSO, THF and dioxane). Thus, pure water remains the optimal solvent.

5. Reproducibility test with different catalyst batches of synthesized Cu/ZrO_2

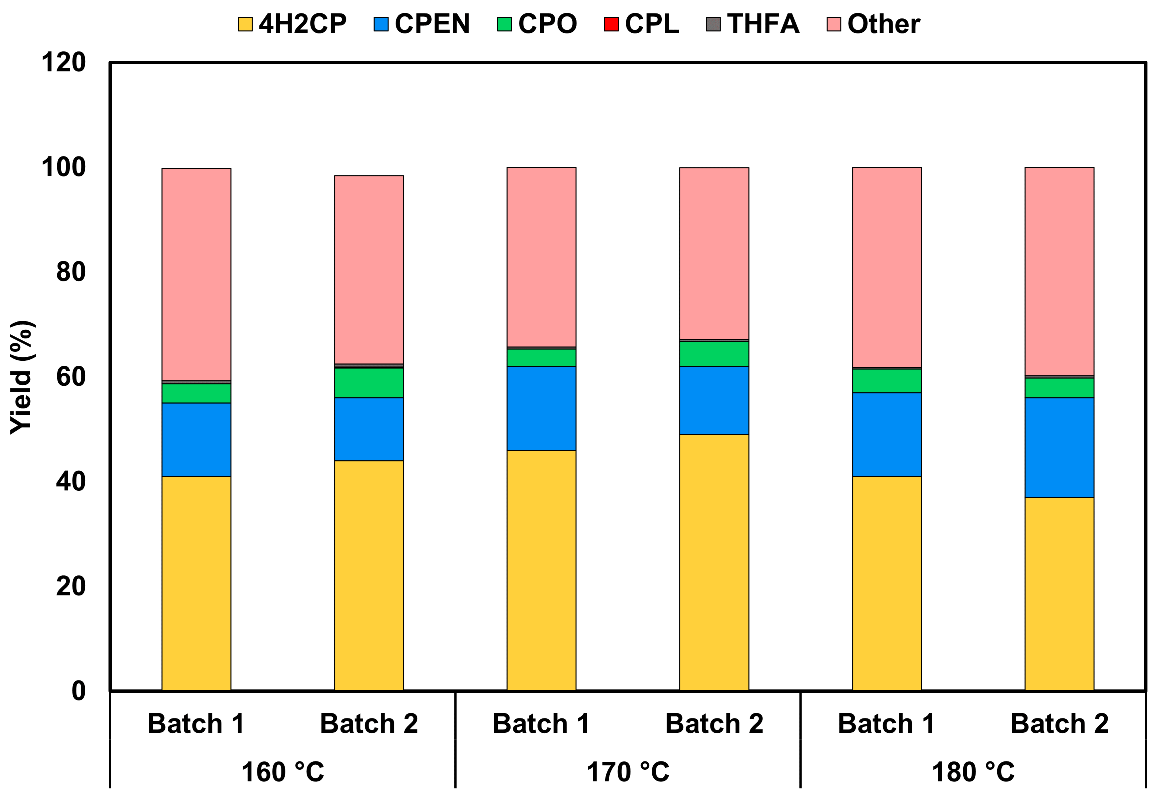

Figure S15. Reproducibility tests of the synthesized Cu/ZrO_2 from different batches using 1 wt% FFA in water. Conditions: T = 160 - 180 °C; P_{total} = 12 bar; W_{cat} = 0.2 grams of Cu/ZrO_2 and β-zeolite each; W_{SiC} = 3.6 gram; Q_{feed} = 0.2 $mL\,min^{-1}$, Q_{H_2}= 1 $NmL\,min^{-1}$; and WHSV = 0.6 $g_{furfural}\,g_{cat}\,hr^{-1}$
